# An Interferon Regulated MicroRNA Provides Broad Cell-Intrinsic Antiviral Immunity through Multihit Host-Directed Targeting of the Sterol Pathway

**DOI:** 10.1371/journal.pbio.1002364

**Published:** 2016-03-03

**Authors:** Kevin A. Robertson, Wei Yuan Hsieh, Thorsten Forster, Mathieu Blanc, Hongjin Lu, Peter J. Crick, Eylan Yutuc, Steven Watterson, Kimberly Martin, Samantha J. Griffiths, Anton J. Enright, Mami Yamamoto, Madapura M. Pradeepa, Kimberly A. Lennox, Mark A. Behlke, Simon Talbot, Jürgen Haas, Lars Dölken, William J. Griffiths, Yuqin Wang, Ana Angulo, Peter Ghazal

**Affiliations:** 1 Division of Pathway Medicine, University of Edinburgh, Edinburgh, United Kingdom; 2 SynthSys at Edinburgh University, The King’s Buildings, Edinburgh, United Kingdom; 3 École Polytechnique Fédérale de Lausanne, Lausanne, Switzerland; 4 Institute of Mass Spectrometry, College of Medicine, Grove Building, Swansea University, Singleton Park, Swansea, United Kingdom; 5 Northern Ireland Centre for Stratified Medicine, University of Ulster, C-Tric, Altnagelvin Campus, Londonderry, Ireland; 6 Centre for Integrative Physiology, Edinburgh, United Kingdom; 7 EMBL – European Bioinformatics Institute, Wellcome Trust Genome Campus, Hinxton, Cambridge, United Kingdom; 8 The Institute of Genetics and Molecular Medicine, Western General Hospital, Edinburgh, United Kingdom; 9 Integrated DNA Technologies, Coralville, Iowa, United States of America; 10 Department of Medicine, University of Cambridge, Cambridge, United Kingdom; 11 Institute of Virology, University of Würzburg, Würzburg, Germany; 12 Institut d’Investigacions Biomèdiques August Pi i Sunyer, Barcelona, Spain; 13 Immunology Unit, Department of Cell Biology, Immunology, and Neurosciences, Medical School, University of Barcelona, Barcelona, Spain; Whitehead Institute, UNITED STATES

## Abstract

In invertebrates, small interfering RNAs are at the vanguard of cell-autonomous antiviral immunity. In contrast, antiviral mechanisms initiated by interferon (IFN) signaling predominate in mammals. Whilst mammalian IFN-induced miRNA are known to inhibit specific viruses, it is not known whether host-directed microRNAs, downstream of IFN-signaling, have a role in mediating broad antiviral resistance. By performing an integrative, systematic, global analysis of RNA turnover utilizing 4-thiouridine labeling of newly transcribed RNA and pri/pre-miRNA in IFN-activated macrophages, we identify a new post-transcriptional viral defense mechanism mediated by miR-342-5p. On the basis of ChIP and site-directed promoter mutagenesis experiments, we find the synthesis of miR-342-5p is coupled to the antiviral IFN response via the IFN-induced transcription factor, IRF1. Strikingly, we find miR-342-5p targets mevalonate-sterol biosynthesis using a multihit mechanism suppressing the pathway at different functional levels: transcriptionally via *SREBF2*, post-transcriptionally via miR-33, and enzymatically via *IDI1* and *SC4MOL*. Mass spectrometry-based lipidomics and enzymatic assays demonstrate the targeting mechanisms reduce intermediate sterol pathway metabolites and total cholesterol in macrophages. These results reveal a previously unrecognized mechanism by which IFN regulates the sterol pathway. The sterol pathway is known to be an integral part of the macrophage IFN antiviral response, and we show that miR-342-5p exerts broad antiviral effects against multiple, unrelated pathogenic viruses such Cytomegalovirus and Influenza A (H1N1). Metabolic rescue experiments confirm the specificity of these effects and demonstrate that unrelated viruses have differential mevalonate and sterol pathway requirements for their replication. This study, therefore, advances the general concept of broad antiviral defense through multihit targeting of a single host pathway.

## Abstract 2

An interferon-induced miRNA suppresses the sterol biosynthesis pathway via multiple targets, thereby helping establish broad cellular resistance to unrelated clinically significant viruses.

## Author Summary

How infected cells respond to a virus during the first minutes to hours after infection can determine whether a disease develops and influences the host’s long-term survival. In mammals, unlike plants and flies that use small RNAs to fight viral infections, virus-induced interferon responses are a critical early event resulting in broad protection against infection. Interferon is a secreted host protein that binds to receptors on the surface of infected and uninfected cells and activates biochemical pathways that profoundly change the expression of hundreds of cellular genes, including those encoding microRNAs. The antiviral functions of only a handful of these genes are understood, and it is not known how the majority contribute to broadly protect against many different viruses. In this study, we uncover an interferon-regulated microRNA (miR-342-5p) that contributes to broad host cell immunity against infection through the cholesterol biosynthesis pathway. We show that miR-342-5p does this through a multihit strategy, turning down the master regulator of sterol biosynthesis as well as several specifically targeted enzymes within the pathway. A wide range of viruses depend on a number of the metabolite side-branches of the sterol biosynthesis pathway for their replication. Notably, our study reveals that by utilising multihit targeting of key branch-points in a single pathway, miR-342-5p is able to inhibit the replication of unrelated, clinically significant pathogens ranging from Herpes to Flu viruses.

## Introduction

**Fig 1:**
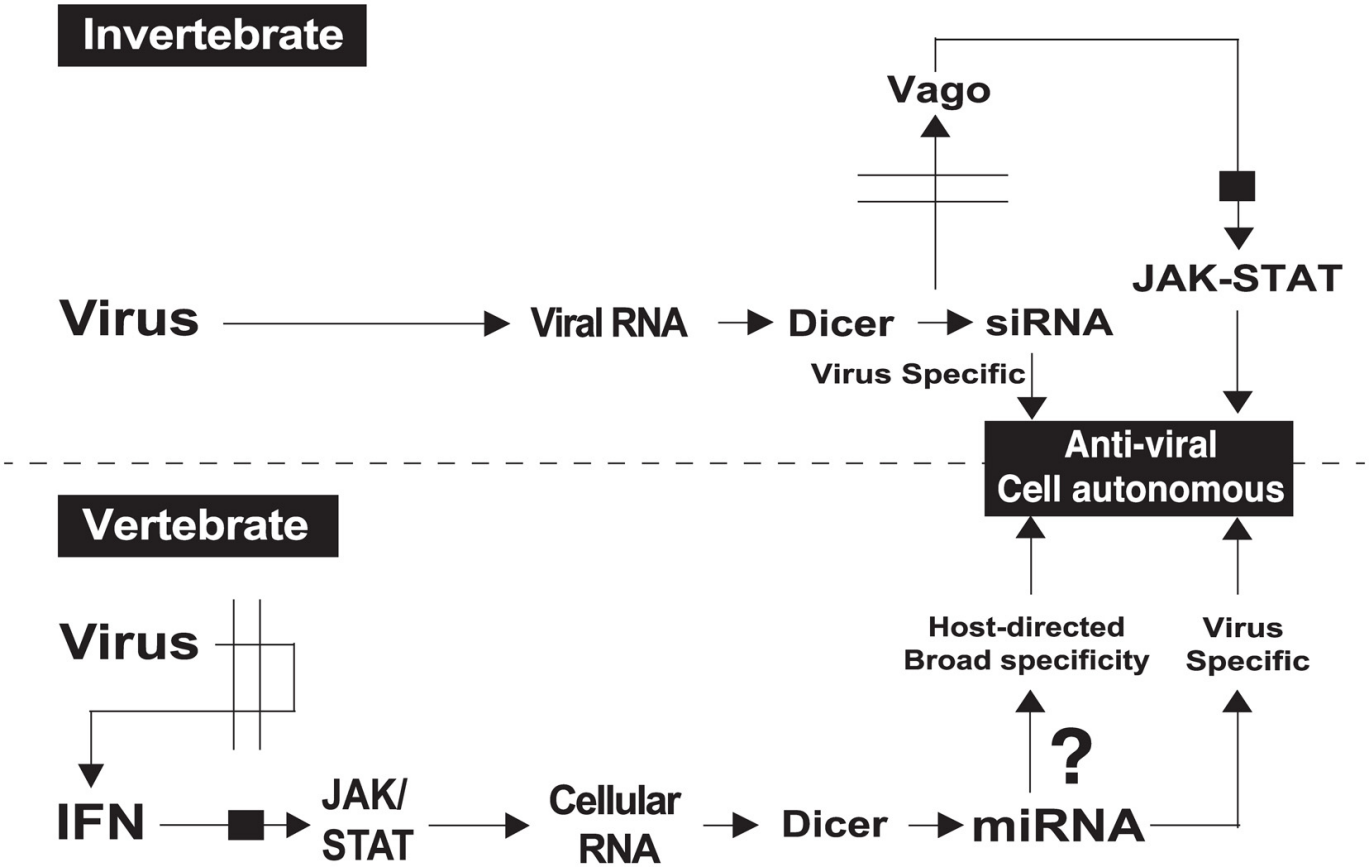
Schematic contrasting antiviral roles for small RNA and IFN-induced miRNA in invertebrates and vertebrates, respectively.

The innate immune response plays a critical role in cellular resistance to infection. In plants and invertebrates, small interfering RNAs (siRNAs) play a vital role in cell-autonomous immunity to viruses. siRNAs are generated and amplified from viral RNA molecules by the cellular RNAi machinery and instil a profound protection against the respective pathogen [1,2]. In mammals, interferon (IFN)-mediated JAK-STAT signalling orchestrates the cellular response to infection and, for several decades, research has focused on the identification and characterisation of antiviral proteins [3]. In contrast, roles for small RNAs in infection of mammalian cells have yet to be fully established (Fig 1) [4].

Several cellular microRNAs have been shown to contribute positively or negatively to infection by targeting host or viral gene expression [5–8]. In this context, IFN stimulation alters the expression of hundreds of cellular genes including some microRNAs [6,9]. Importantly, however, antiviral functions have been attributed to very few of these, although evidence for IFN-induced miRNAs targeting specific pathogens has been documented [6]. A key unresolved issue with important therapeutic implications is: how can IFN-regulated miRNA enhance antiviral functionality? In 2007, Pedersen et al. showed that an IFN-elicited down-regulation of a virus-targeting host miRNA (miR-122) leads to a reduction in Hepatitis C virus replication. This was notable as, historically, studies of IFN antiviral effects have focused on induced genes and this work, identifying a reduction in the expression of a virus-targeting host miRNA, has led to significant clinical advances [6,10]. Attention has also focused on interferon-regulated miRNA enhancement of IFN-mediated antiviral effects through the suppression of negative regulators such as the SOCS proteins [11]. Importantly, whilst antiviral miRNAs targeting host transcripts are emerging, few, if any, studies have characterised the precise mechanisms through which these function. miRNA targeting of multiple transcripts opens up potential for the simultaneous regulation of divergent and convergent cellular pathways and functions. It still remains an open question whether mammalian IFN-induced host-directed miRNAs act as effectors of the cell-intrinsic antiviral immune response. Such a strategy would arguably confer an advantage, as it has the potential to endow a broad-spectrum antiviral activity and greater resistance to the development of escape mutants.

Viruses rely on metabolic and biosynthetic cellular pathways for their replication, and a common feature is a dependency on cellular lipid metabolism [12]. As such, pharmacological inhibition of lipid biosynthesis can curtail virus replication [13–19]. Moreover, there is increasing evidence that the immune system and lipid pathways are tightly coupled [20–22] and defining how innate immunity and lipid metabolism are integrated, share resources, and crossregulate one-another during infection may result in new therapeutic strategies [20,23–26]. In this regard, IFN-induced inhibition of sterol biosynthesis serves as an integral component of the very early cellular response to virus infection and we, and others, have shown that cholesterol 25-hydroxylase (CH25H) and its cognate metabolite 25-hydroxycholesterol (25-HC) are important effectors in this response [18,24,27].

In this study, we sought to further unravel a previously unreported cellular mechanism underpinning immune regulation of lipid pathways and the inhibition of a broad range of viruses. We present evidence for a new miRNA-mediated cell-intrinsic antiviral effector arm of the IFN response, activated during the very early stages of infection. The miRNA under investigation has the capacity to instil a broad antiviral state in vitro and in vivo. Mechanistically, we present data to show the broad antiviral functions of the miRNA occur via a specific multihit suppression of the sterol-biosynthetic pathway.

## Results

### IFN Uses Multiple Mechanisms to Suppress Sterol Biosynthesis

**Fig 2:**
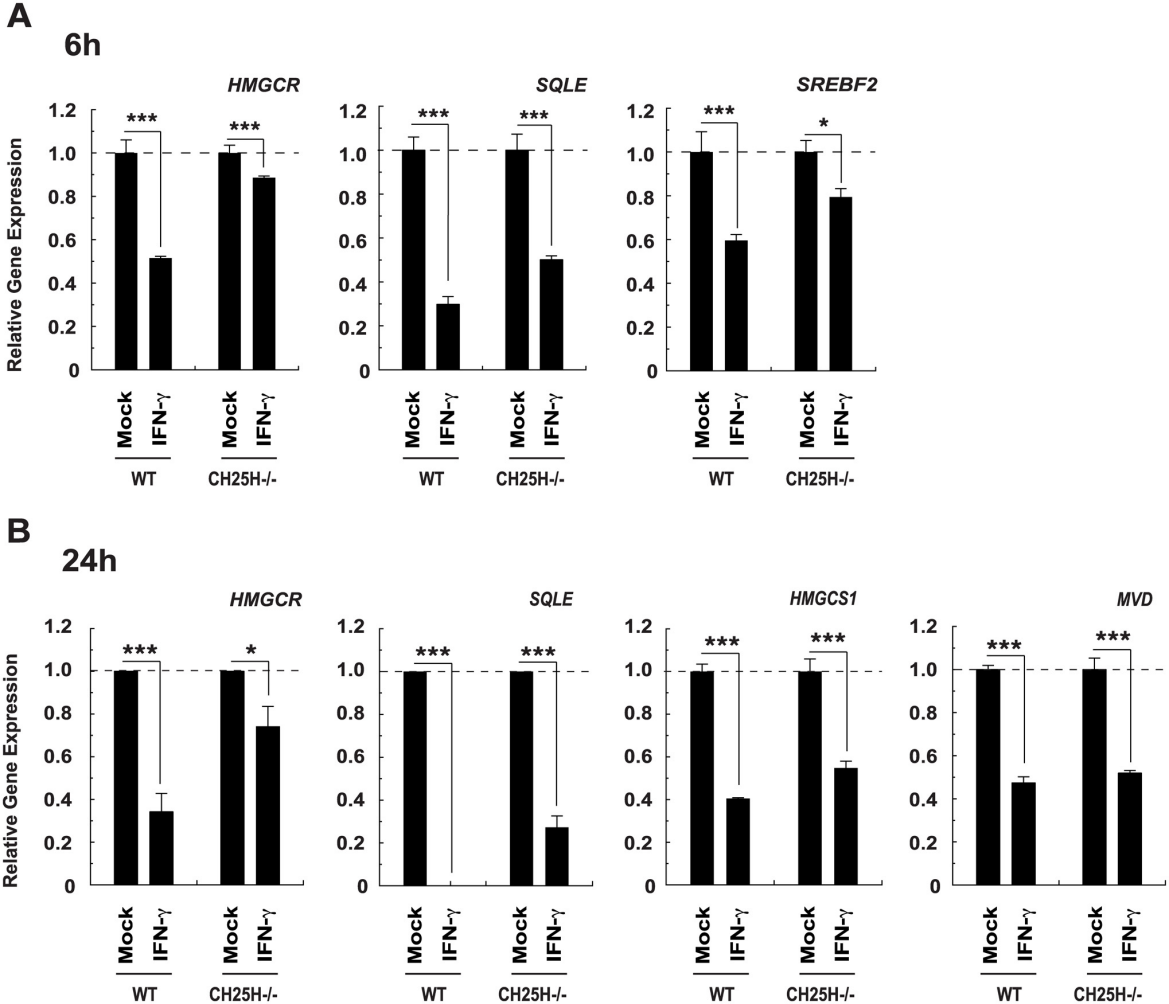
IFN-γ treatment of CH25H^-/-^ BMDM decreases the abundance of cholesterol biosynthesis pathway transcripts. (A) *HMGCR*, *SQLE*, and *SREBF2* transcript abundance in bone marrow-derive macrophage (BMDM) derived from wild-type or CH25H^-/-^ mice and treated with IFN-γ (10 U/ml) for 6 h. All data are mean +/− standard error of the mean (SEM). *n* = 3, *** *p* ≤ 0.001 by 1-sample *t* test. (B) *HMGCR*, *SQLE*, *HMGCS1*, and *MVD* transcript abundance in BMDM derived from wild-type or CH25H^-/-^ mice and treated with IFN-γ (10 U/ml) for 24 h. All data are mean +/− SEM. *HMGCS1* data *n* = 3, *** *p* ≤ 0.001 by 1-sample *t* test. *HMGCR* data *n* = 9, * *p* ≤ 0.05, *** *p* ≤ 0.001 by 1-sample *t* test. *MVD* data, *n* = 3, *** *p* ≤ 0.001 by 1-sample *t* test. Squalene (SQL) data, wild-type BMDM *n* = 3: IFN-γ *** *p* ≤ 0.001 by 1-sample *t* test, CH25H^-/-^ BMDM, *n* = 6, *** *p* ≤ 0.001 by 1-sample *t*-test.

In experiments, seeking to address whether the activity of CH25H (UniProt: Q9Z0F5) was sufficient to account for the IFN-induced down-regulation of cholesterol biosynthesis in macrophages, we measured the abundance of several key sterol pathway transcripts including *HMGCS1* (Entrez Gene: 208715), *HMGCR* (Entrez Gene: 15357), *MVD* (Entrez Gene: 192156), *SQLE* (Entrez Gene: 20775), and *SREBF2* (Entrez Gene: 20788) in *CH25H* ^-/-^ cells treated with interferon gamma (IFN-γ) (UniProt: P01580). Importantly, in the absence of CH25H, the effects of IFN-γ on these transcripts were reduced but not abrogated (Fig 2A and 2B) at 6 h (*HMGCR*, *SQLE and SREBF2*) and 24 h (*HMGCS1*, *HMGCR*, *MVD and SQLE*) after addition of the cytokine. These data suggested that 25-HC-dependent and independent mechanisms are involved early in the suppressive effect of IFN-γ on the sterol pathway.

**Fig 3:**
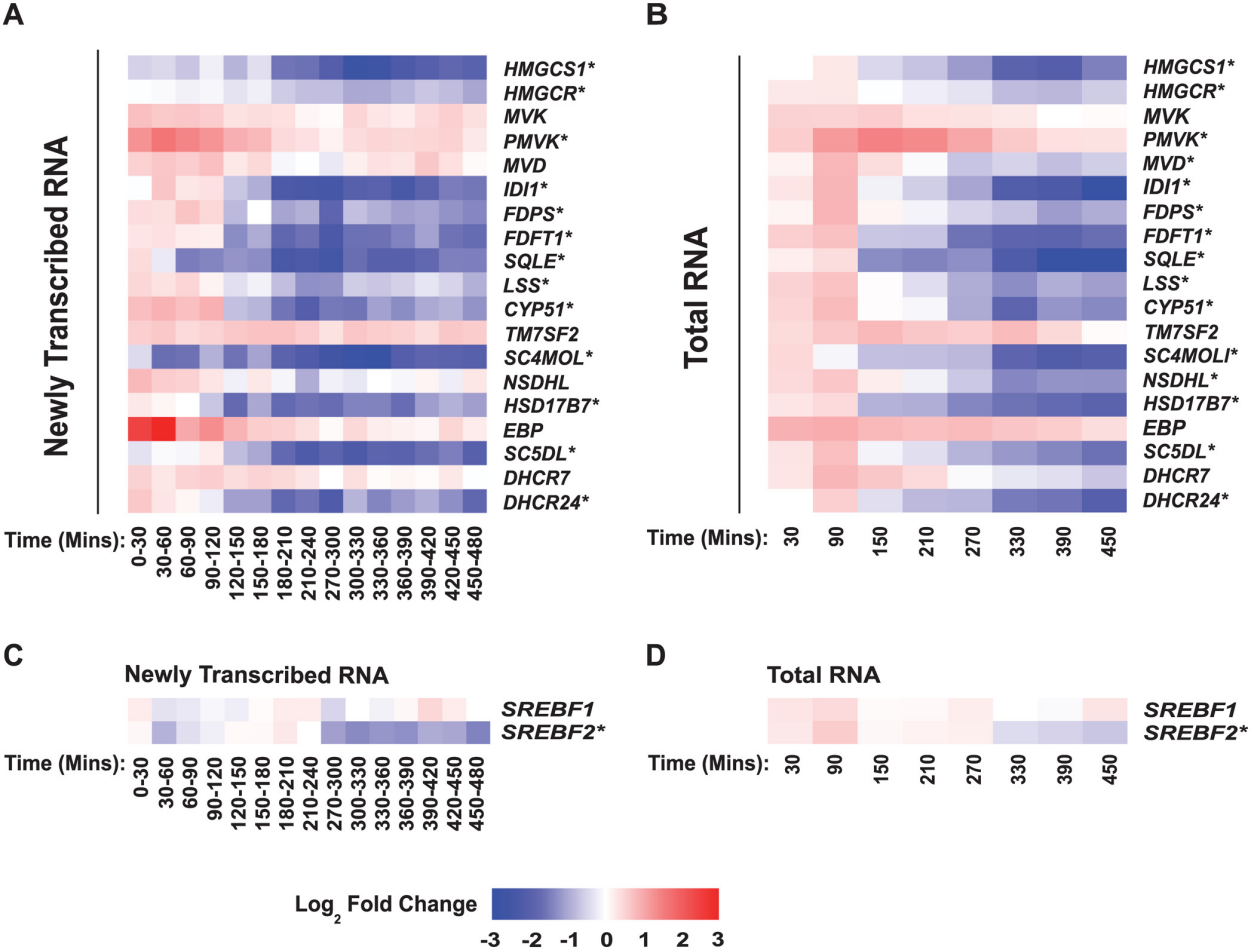
IFN-γ treatment of macrophages decreases the synthesis and abundance of cholesterol biosynthesis pathway transcripts. (A) Sequential analysis of sterol pathway-related transcript synthesis (every 30 min) in IFN-γ treated BMDM (relative to mock). (B) Abundance of sterol biosynthesis pathway-related transcripts in IFN-γ treated BMDM (relative to mock). (C) Synthesis of *SREBF1* and *2* transcripts in IFN-γ treated BMDM (relative to mock). (D) Abundance of *SREBF1* and *2* transcripts in IFN-γ treated BMDM (relative to mock). In Fig 3A, 3B, 3C, and 3D, each column represents a time period and each row one gene. Gene expression is shown as a pseudocolour: blue = decrease, red = increase. Log_2_ fold change values were calculated by subtracting the Mock from the IFN-γ treated Log_2_ scale signal value. * = Significant by MaSigPro test (see Materials and Methods and S1 Methods).

**Fig 4:**
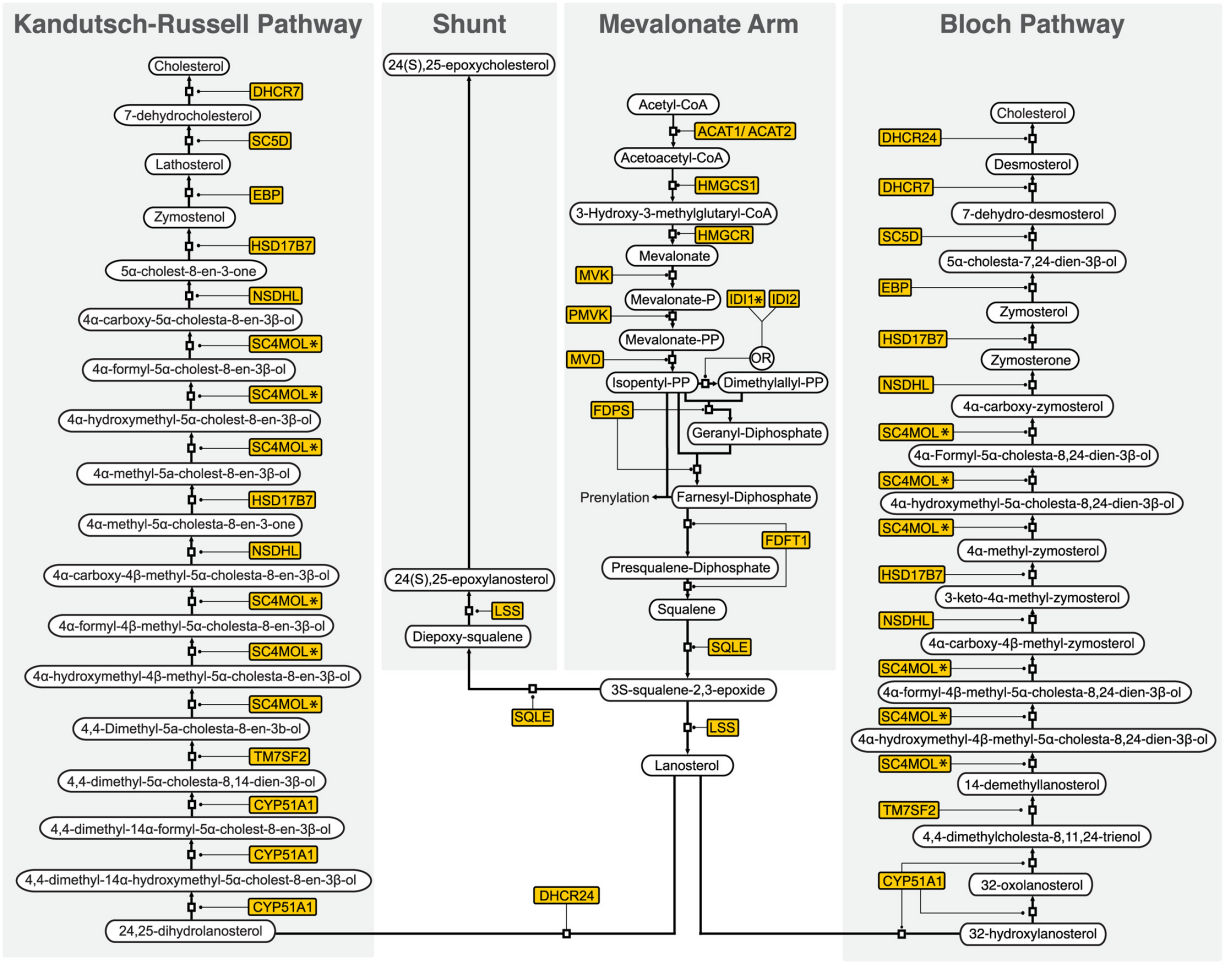
Schematic of the cholesterol biosynthesis pathway adapted from Mazein et al. (2013) [28]. * indicates pathway targets for miR-342-5p

To gain further kinetic insights into IFN-γ-elicited alterations in mRNA expression, we undertook a serial (every 30 mins for 8 h) high-resolution, systematic microarray analysis of de novo RNA synthesis and overall RNA abundance in bone marrow-derived macrophages (BMDM) treated with IFN-γ. A flow diagram summarising our protocol for the isolation and parallel analysis of newly transcribed and total RNA is presented (S1 Fig). These experiments revealed a significant down-regulation in the rate of transcript synthesis for 13/19 members of the cholesterol pathway (Data: Fig 3A and 3B and S2, S3 Figs Pathway: Fig 4) [28].

Decreases in the rate of pathway transcript synthesis commenced 2 h to 3 h after treatment (Fig 3A, S2 Fig) and were maintained for the duration of the time course (8 h). In addition, the rate of synthesis and abundance of *SREBF2* (but not *SREBF1* (Entrez Gene: 20787) also decreased in response to IFN-γ treatment (Fig 3C and 3D, S2 and S3 Figs). We note a more pronounced repression of *SREBF2* synthesis over abundance, which likely reflects an IFN-γ action on the well-characterised transcriptional autoregulation of SREBP2 [29]. Taken together, this data is consistent with previous studies [18,24,30,31], demonstrating that transcriptional mechanisms play a role in IFN-γ-mediated down-regulation of cholesterol. However, unexpected reductions in the abundance, but not synthesis of transcripts such as *MVD*, *NSDHL*, and *DHCR7* (Fig 3A and 3B, S2 and S3 Figs) suggested that cholesterol pathway transcripts may also be subject to 25-HC-independent post-transcriptional regulation. As IFN-γ-mediated suppression of the sterol pathway is strictly dependent on JAK-STAT signalling, we hypothesised that a likely post-transcriptional mechanism might involve IFN-stimulated miRNAs specifically targeting transcripts within the sterol metabolic network.

### IFN Is Coupled to miR-342 Expression

**Fig 5:**
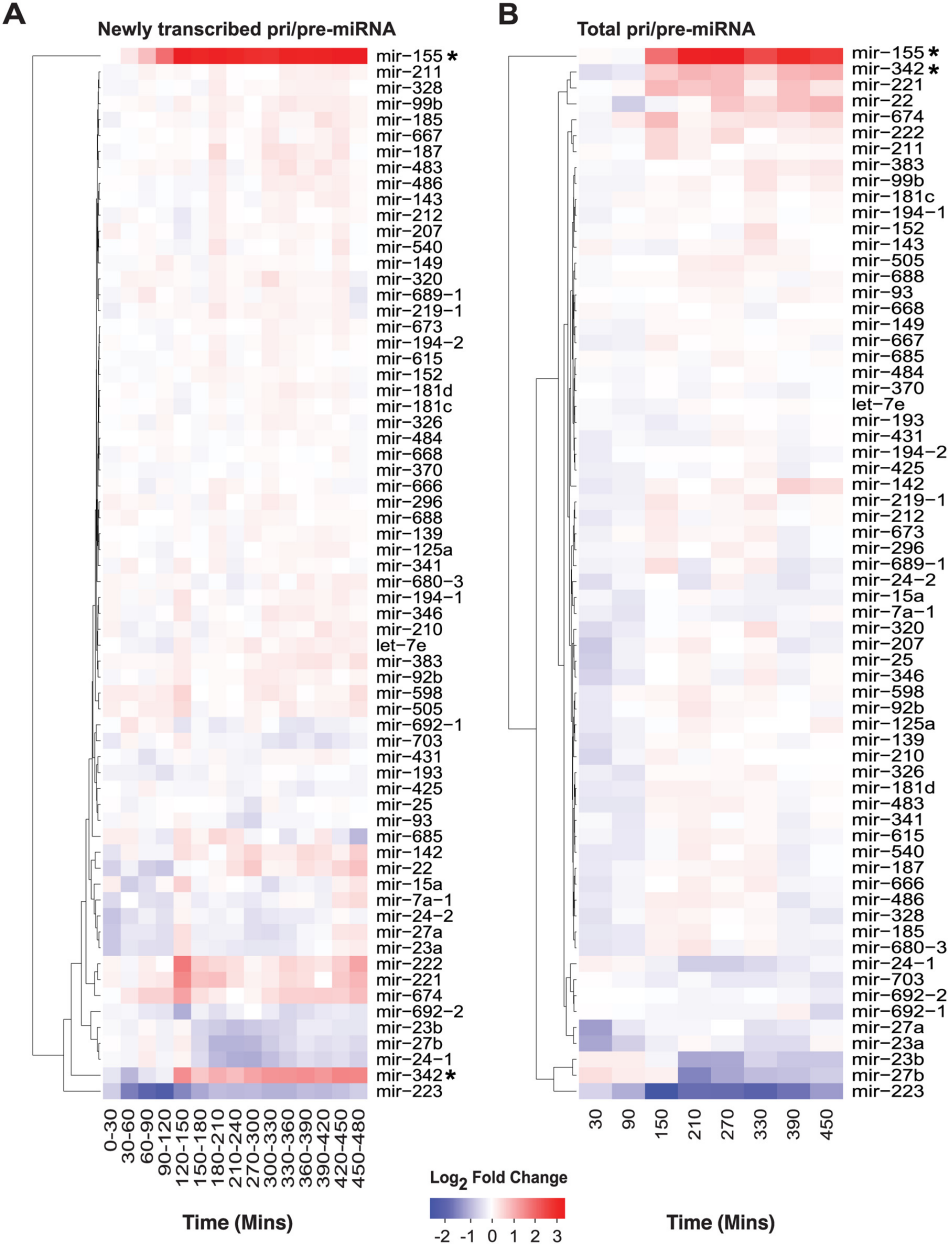
MiR-155 and miR-342 synthesis and abundance are significantly up-regulated in IFN-γ treated BMDM. (A and B) Pri/pre-miRNA synthesis (A) and abundance (B) in BMDM following IFN-γ stimulation (10 U/ml). Timecourse data are represented as described for Fig 3. * = Significant by MaSigPro test (see Materials and Methods and S1 Methods).

**Fig 6:**
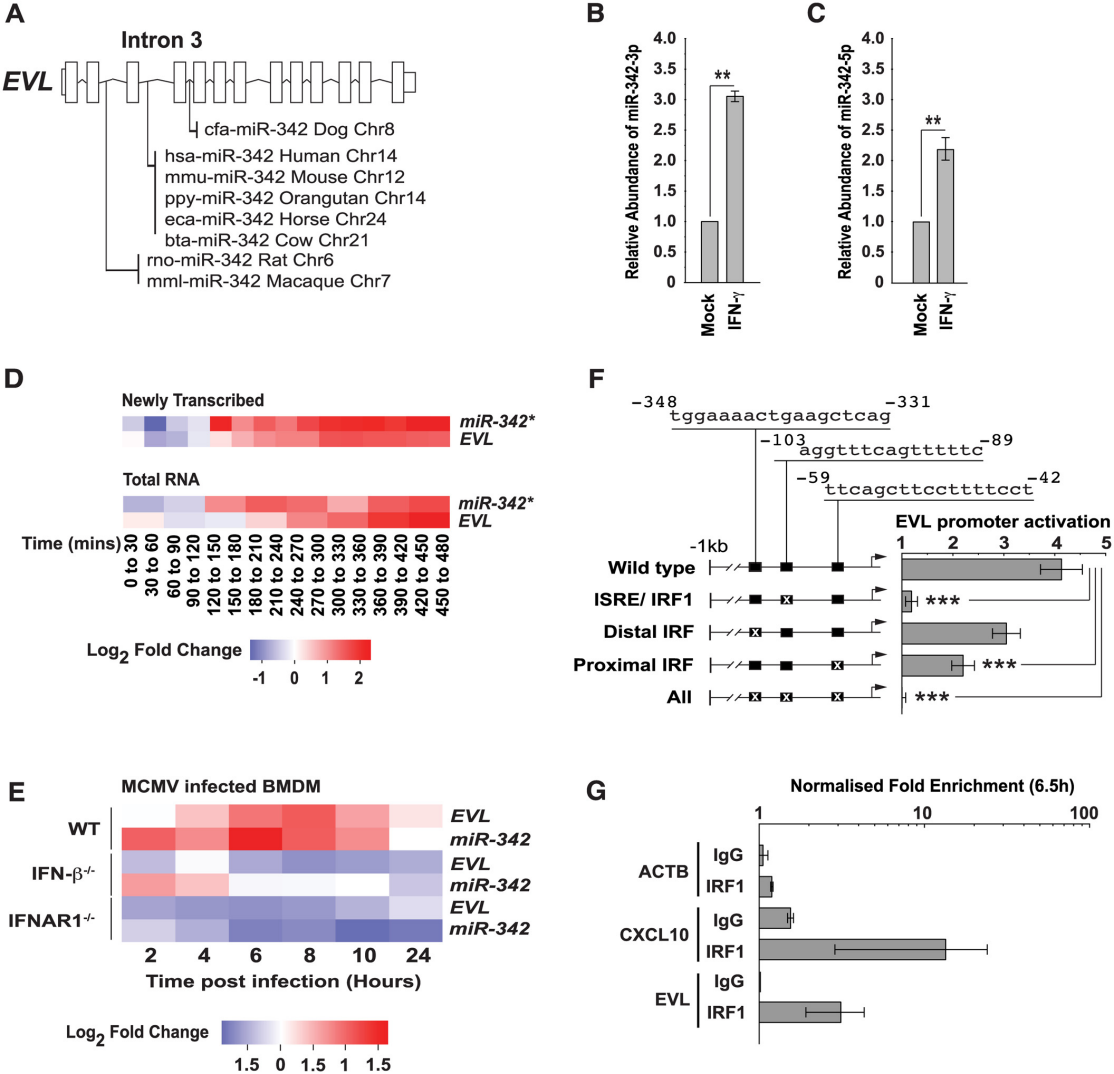
MiR-342 expression is directly coupled to IFN. (A) MiR-342 is encoded in the gene EVL and conserved between species. (B) Mature miRNA-342-3p expression relative to a small nucleolar RNA (SNORD47) in BMDM 24 h after treatment with 10 U/ml IFN-γ. Data are mean +/− SEM (*n* = 3). ** *p* ≤ 0.01 by 1-sample *t* test. (C) Mature miRNA-342-5p expression relative to a small nucleolar RNA (SNORD47) in BMDM 24 h after treatment with 10 U/ml IFN-γ. Data are mean +/− SEM (*n* = 10, ** *p* ≤ 0.01). (D) *EVL* and pri/pre-miR-342 RNA transcript synthesis and abundance in BMDM following IFN-γ stimulation (10 U/ml). Time course data are represented as described for Fig 1. * = Significant by MaSigPro test (see Materials and Methods and S1 Methods). (E) EVL and pri/pre-miR-342 (relative to *t* = 0) abundance in WT, IFNB1−/− or interferon alpha receptor 1 (IFNAR1−/−) BMDM infected with murine cytomegalovirus (MCMV) (multiplicity of infection [MOI] = 1). (F) Wild type or mutated 1 kb *EVL* promoter regions upstream of a luciferase reporter were used to test the relative importance of predicted ISRE, distal interferon regulatory factor (IRF), proximal IRF (or all) binding sites on IFN-induced expression (*n* = 10, *** *p* ≤ 0.001). (G) Chromatin immunoprecipitation analysis of IRF1 transcription factor binding to *ACTB* (negative control), *CXCL10* (positive control), or the *EVL* promoter in BMDM cultured with IFN-γ (10 U/ml) for 6.5 h. Data are mean +/− SEM, *n* = 2.

In support of the above hypothesis, studies have documented miRNA which can regulate lipoprotein uptake (e.g., miR-125a and -455), lipid biosynthetic enzyme expression (e.g., miR-155, miR-21, and miR-185) and, in particular, cholesterol efflux (e.g., miR-33, miR-144) [32–37]. IFN-γ treatment of a melanoma cell line suggested that some of these miRNA (e.g., miR-125a, -455 and -185) may be part of an IFN response; however, it is not known if they are directly coupled to IFN [9]. Thus, we assessed changes in the expression of miRNA precursors (pri/pre-miRNAs) in BMDM stimulated with IFN-γ. Using conservative criteria for detection, we identified 66 pri/pre-miRNAs in our macrophage data (Fig 5A and 5B). Temporal analysis of our time course microarray data, however, revealed two of these, namely pri/pre-miR-155 (Entrez Gene: 387173) and pri/pre-miR-342 (Entrez Gene: 723909), to be significantly up-regulated during the first 8h of IFN-γ treatment (Fig 5A and 5B). MiR-155 is an evolutionarily highly conserved, NF-κB-responsive miRNA encoded by the MIR155 host gene (MIR155HG). It is highly expressed in activated macrophages and lymphocytes [38]. Much less is known about the conserved miR-342 located in an intron of the Ena-vasodilator stimulated phosphoprotein gene (*EVL–*Entrez Gene: 14026) in the mouse or Ena-Vasp-Like (*EVL–*Entrez Gene: 51466) in the human (Fig 6A) [39]. Gene expression data from 89 mouse cells and tissues analysed in the BioGPS GeneAtlas indicates the *EVL* transcript is primarily expressed in cells of the immune and nervous system [40]. In macrophages, miR-342 has previously been identified as a PU.1-regulated miRNA contributing to myeloid differentiation and a proinflammatory mediator capable of enhancing miR-155 expression [41,42]. To confirm the presence of mature miR-155, miR-342-3p and -5p derived from the precursors detected in our array analysis, we stimulated BMDM with IFN-γ (10 U/ml) or interferon beta (IFN-β) (Uniprot: P01575) (25 U/ml) and analysed miRNA expression using quantitative reverse transcription polymerase chain reaction (Q-RT-PCR). In these analyses, significant increases in the expression for all 3 miRNAs were observed (Fig 6B and 6C, S4A and S4B Fig).

Fig 6D and S4C Fig show the coordinate regulation of both the synthesis and abundance of the primary *EVL* transcript and pri/pre-miR-342 in IFN-γ stimulated BMDM. Both the primary transcript and precursor decreased in synthesis rate (relative to the mock) in the first 60 min after treatment. This was followed by an elevated synthesis rate and increased abundance from around 120 min onwards. Although the transcription factors IRF1 (UniProt: P10914) and IRF9 (UniProt: Q00978) have been implicated in the regulation of *EVL* in the context of long-term retinoic acid treatment of human promyelocytic leukaemia cells [43], it is not known whether the *EVL* promoter can be directly regulated by IFN and whether IFN signalling alone is sufficient for its induction [42]. To test the cell-autonomous requirement for IFN signalling in the up-regulation of *EVL* and pri/pre-miR-342 during infection, we analysed by microarray the endogenous regulation of these transcripts in BMDM with a genetic ablation in IFN beta (IFN-β), the type 1 IFN receptor (IFNAR1 –UniProt: P17181) (Fig 6E) or the down-stream signalling molecule tyrosine kinase 2 (TYK2 UniProt: Q9R117) (S4D and S4E Fig). Ablation of IFN signalling abolished murine cytomegalovirus (MCMV)-induced induction of EVL and pri/pre-miR-342 in all cases demonstrating that virus-induced up-regulation of EVL and pri/pre-miR-342 is dependent on an intact type 1 IFN response. By computational promoter analysis, we identified three putative binding motifs (one IFN-stimulated response element/IRF1 binding site [ISRE/IRF1] and two potential IFN regulatory factor [IRF] binding sites) within a 1 kb region of the *EVL* promoter (S5A Fig). Luciferase reporter plasmids containing a wild-type promoter or promoters with one or multiple mutations in the three predicted motifs validated the ISRE/IRF1 motif (Fig 6F). Mutation of either the proximal or distal interferon regulatory factor (IRF) motifs resulted in an intermediate reduction in promoter activity. We next performed chromatin immunoprecipitation assays to test for STAT1 (UniProt: P42225) and/or IRF1 (UniProt: P15314) binding to the murine *EVL/*miR-342 promoter. In IFN-γ-treated murine BMDM, the positive control CXCL10 promoter, but not the EVL/mir-342 promoter, was occupied by STAT1 or IRF1 2 h after treatment (S5B Fig upper graph). By 6.5 h, however, we observed an IRF1-specific enrichment (~3-fold) of *EVL/*miR-342 promoter sequences (Fig 6G) and by 24 h, IRF1 recruitment was still detectable (S5B Fig lower graph). We conclude that canonical IFN signalling is required for increased expression of miR-342 and that this involves the recruitment of IRF1 to the *EVL/*miR-342 promoter.

### MiR-342-5p Regulates Sterol Biosynthesis

**Fig 7:**
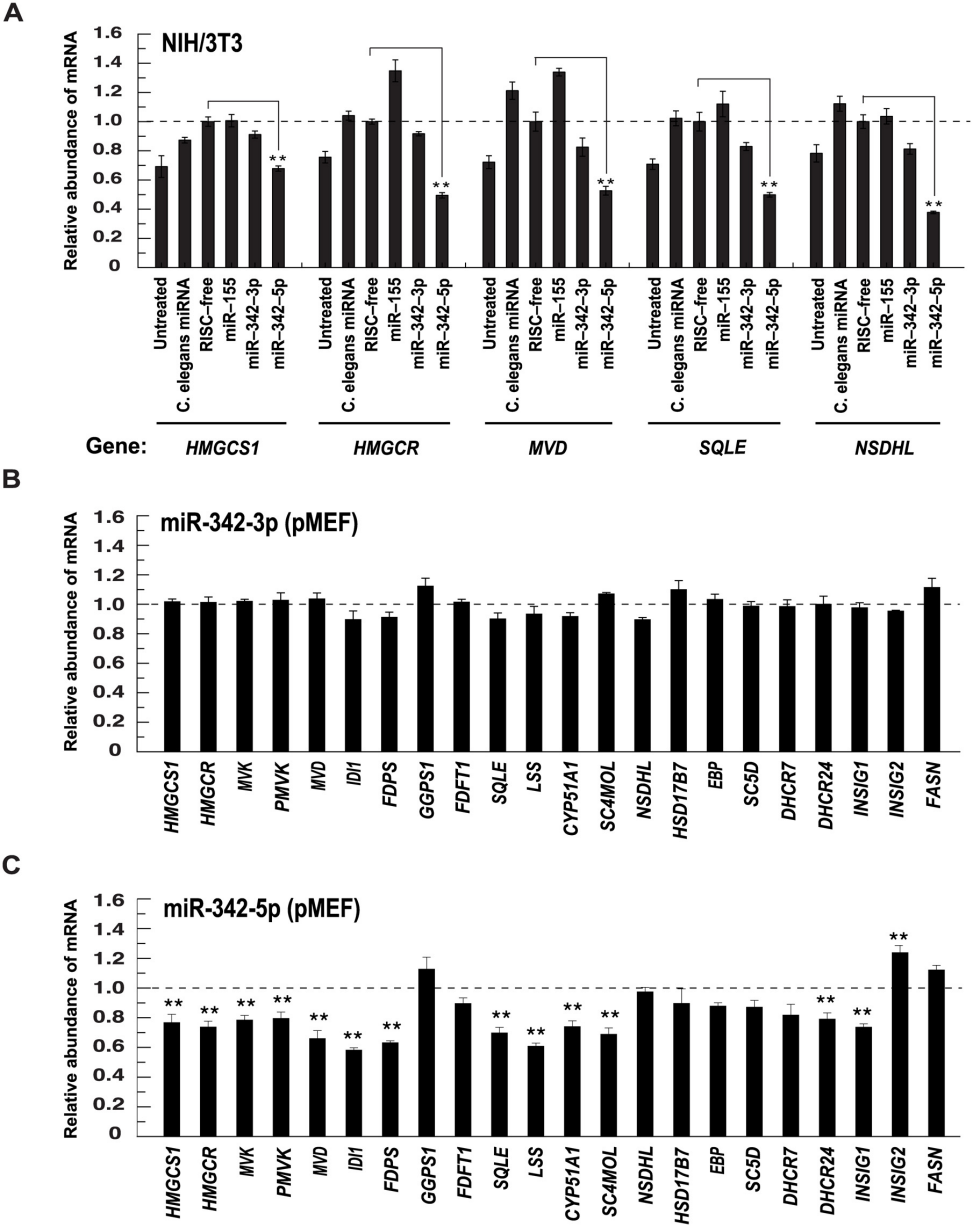
MiR-342-5p regulates the sterol pathway. (A) IFN-regulated miRNA effects on cholesterol pathway transcript abundance in NIH/3T3 fibroblasts. Data normalised to nontargeting siRNA and are mean +/− SEM (*n* = 3). ** *p* ≤ 0.01. (B) MiR-342-3p effects on cholesterol pathway transcript abundance in pMEF. Data are normalised to nontargeting siRNA and are mean +/− SEM (*n* = 3). (C) MiR-342-5p effects on cholesterol pathway transcript abundance in pMEF. Data are normalised to nontargeting siRNA and are mean +/− SEM (*n* = 6), ** *p* ≤ 0.01.

MiR-342 has recently been implicated in the regulation of SREBP2 in a cancer cell line; however, biological roles and precise mechanisms for the miRNA in relation to sterol biosynthesis and the immune response were not addressed [44]. To systematically test whether miR-155 or miR-342 could regulate the cholesterol pathway, miR-155, -342-3p or -342-5p mimics were transfected into a fibroblast cell line and RNA levels for selected cholesterol biosynthesis enzymes quantitated. While miR-155 and miR-342-3p had no significant effect on gene expression in the cholesterol pathway, miR-342-5p reduced the overall abundance of *HMGCS1* (−32%), *HMGCR* (−51%), *MVD* (−48%), *SQLE* (−51%) and *NSDHL* (Entrez Gene: 18194) (−62%) relative to cells treated with the nontargeting control (Fig 7A). In a comprehensive sterol pathway analysis of RNA extracted from primary murine embryo fibroblasts (pMEF) transfected with miR-342-3p or -5p, we proceeded to show that miR-342-5p but not miR-342-3p induced a significant, coordinate reduction in the abundance of 12 out of 19 sterol biosynthesis pathway transcripts analysed (Fig 7B and 7C). S6 Fig shows a comparison of the reduction in abundance of the sterol pathway transcripts by miR-342-5p with that elicited by a *SREBF2* targeting siRNA. Notably, the abundance of the fatty acid-associated transcript *FASN* (Entrez Gene: 14104) was not altered by miR-342-5p in these experiments (Fig 7B and 7C).

**Fig 8:**
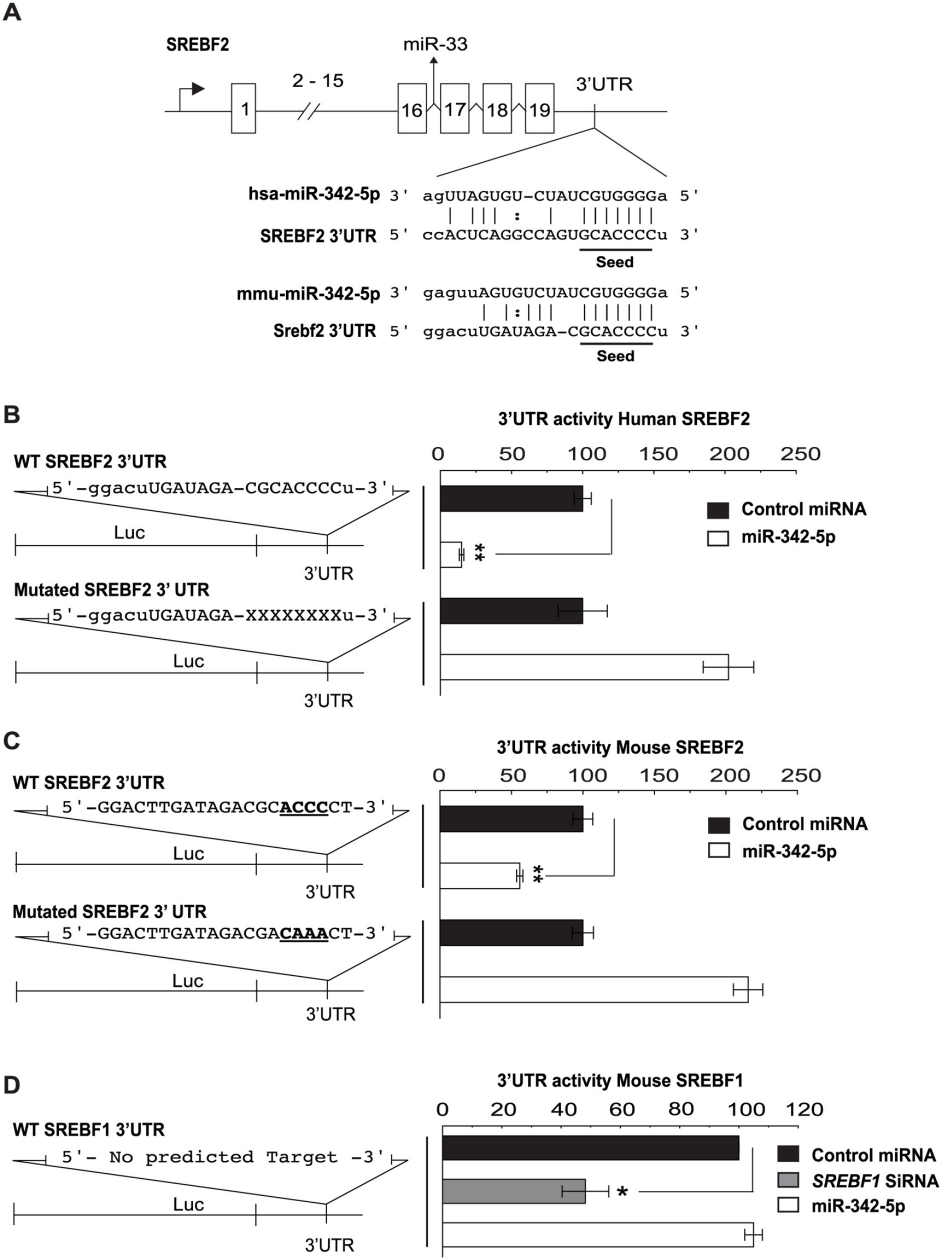
MiR-342-5p specifically targets the *SREBF2* transcript. (A) Predicted targets for miR-342-5p in human and mouse *SREBF2* transcripts. (B) Specific targeting of Human *SREBF2* 3’UTR by miR-342-5p. Data are mean +/− SEM (*n* = 3). ** *p* ≤ 0.01. (C) Specific targeting of Mouse *SREBF2* 3’UTR by miR-342-5p. Data are mean +/− SEM (*n* = 3). ** *p* ≤ 0.01. (D) MiR-342-5p does not target wild-type 3’UTR of murine SREBF1. Data are mean +/− SEM (*n* = 3). * *p* ≤ 0.05.

**Fig 9:**
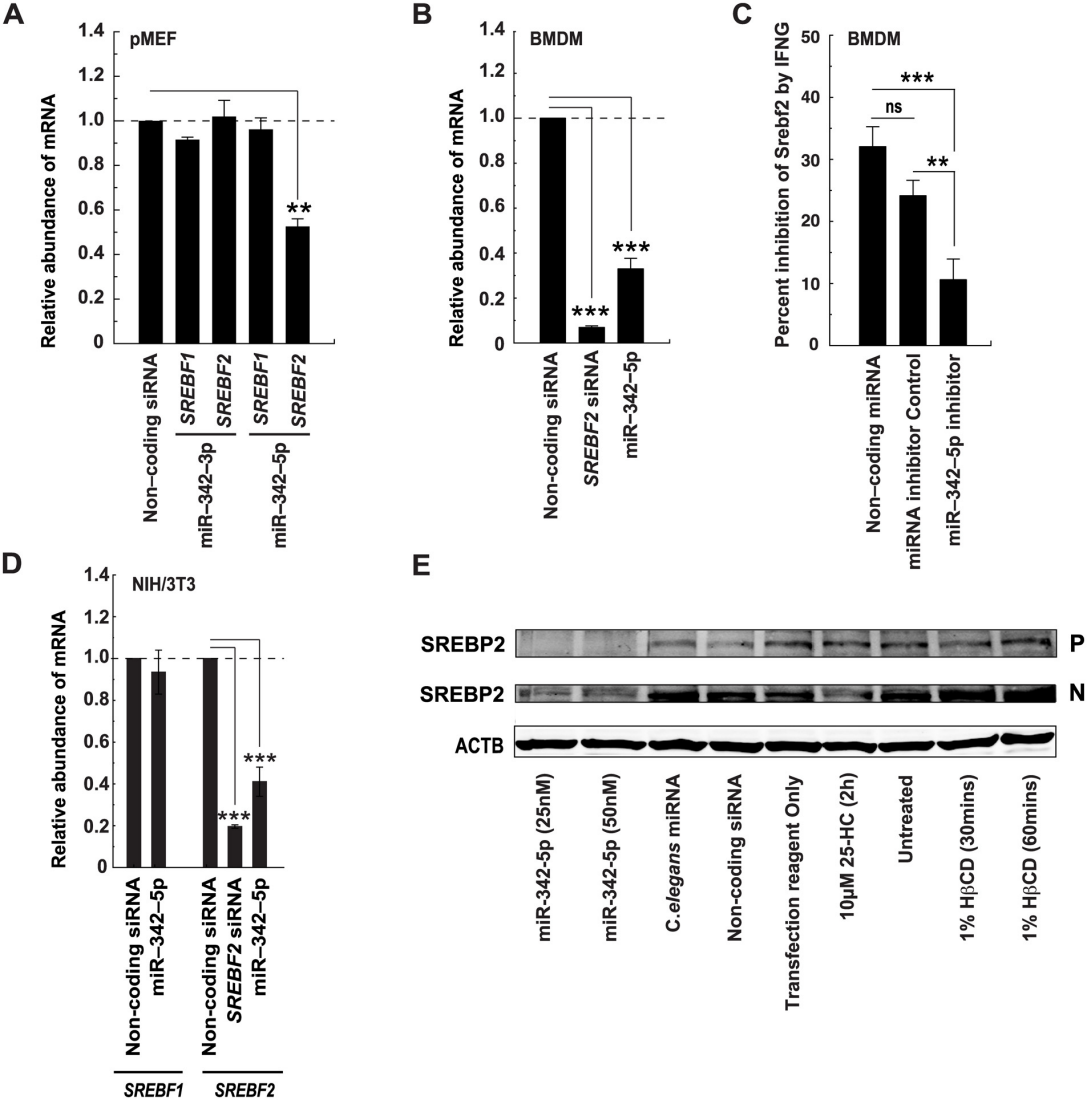
MiR-342-5p regulates SREBF2/SREBP2. (A) MiR-342-3p and -5p effects on *SREBF1* and *SREBF2* transcript abundance in pMEF. Data are mean +/− SEM (miR-342-3p *n* = 3, miR-342-5p *n* = 6), ** *p* ≤ 0.01). (B) MiR-342-5p effects on *SREBF2* transcript abundance in BMDM. Data are mean +/− SEM (*SREBF2* siRNA *n* = 5, miR-342-5p *n* = 6). *** *p* ≤ 0.001. (C) A miR-342-5p inhibitor reduces the effects of IFNG-elicited *SREBF2* repression in BMDM. IFNG treatment was 2.5 ng/ml for 24 h; data are mean +/− SEM (*n* = 6 except SREBF2 siRNA *n* = 5). ** *p* ≤ 0.01, *** *p* ≤ 0.001. (D) MiR-342-5p effects on SREBF1 and SREBF2 transcript abundance in NIH/3T3 fibroblasts. Data normalised to nontargeting siRNA and are mean +/− SEM (SREBF2 siRNA *n* = 6, miR-342-5p *n* = 9). ** *p* ≤ 0.001 by 1-sample *t* test. (E) Western blot analysis of Uncleaved (P) or cleaved (N) SREBP2 protein changes due to miR-342-5p, *Caenorhabditis elegans* miRNA, RISC-free siRNA, Transfection Reagent Only, 10 μM 25-HC for 2 h, 1% HβCD for 30 min or 1% HβCD for 60 min in NIH/3T3 cells.

The concordant reduction of so many genes was consistent with the repression of a master regulator of the sterol biosynthesis pathway. SREBP2, the master transcriptional regulator for the majority of genes encoding sterol pathway enzymes, contains a predicted miR-342-5p binding site that is conserved in both human and mouse (Fig 8A). To directly test the function of this predicted miR-342-5p target, we generated dual-luciferase reporter constructs for the human and mouse *SREBF2* and mouse *SREBF1* 3’UTRs. Cotransfection of these reporters with miR-342-5p significantly reduced luciferase activity for the murine *SREBF2* but not the *SREBF1* reporter construct (Fig 8B, 8C and 8D). In addition, mutation of the seed region in both the human and mouse *SREBF2* 3’UTR increased luciferase expression (Fig 8B and 8C). Transfection of several mouse or human cell types with the miR-342-5p mimic decreased *SREBF2* transcript abundance (Fig 9A: pMEF, Fig 9B: BMDM, Fig 9D: NIH/3T3, S7A and S7B Fig). To avoid any complications arising from miRNA overexpression, we also sought to test the effect of inhibiting endogenous miR-342-5p on IFNG-mediated *SREBF2* regulation. In these experiments, we found miR-342-5p inhibitor significantly decreased but did not eliminate the repressive effects of a nonsaturating dose of IFNG [45] on *SREBF2* copy number in BMDM (Fig 9C). The results of these investigations show a contributory role of approximately 30% to 50% for miR-342-5p in the macrophage IFN suppression of *SREBF2*.

In further experiments, protein levels for both the uncleaved (P) and cleaved (N) form of the SREBP2 (UniProt: Q3U1N2) protein were also decreased in cells transfected with the miR-342-5p mimic (Fig 9E). The latter result contrasts with the action of 25-HC that reduced the cleaved protein (N) abundance alone (Fig 9E). Human *SREBF1* (Entrez Gene: 6720) has recently been identified as a target of miR-342-5p (confirmed in S7B Fig) [44]. Our bioinformatics analysis did not identify a target for this miRNA anywhere in the murine transcript, and the transfection of mouse fibroblasts with a miR-342-5p mimic did not elicit a significant decrease in overall SREBF1 transcript abundance (Fig 9A and 9D).

**Fig 10:**
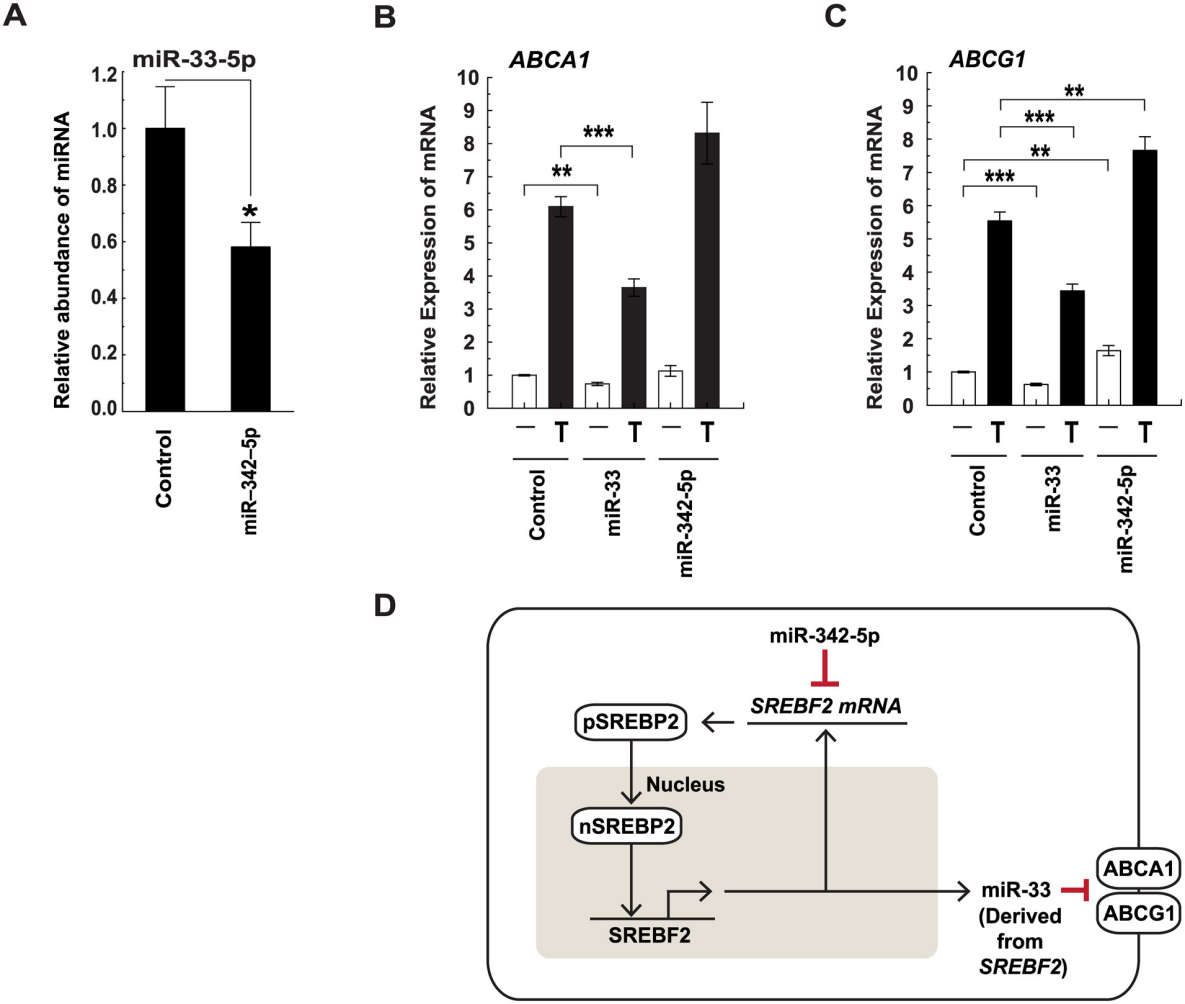
MiR-342-5p regulates miR-33 expression and function. (A) MiR-342-5p effects on miR-33-5p abundance. Data are mean +/− SEM (*n* = 3) * *p* ≤ 0.05 by 1-sample *t* test. (B) MiR-342-5p effects on *ABCA1* transcript abundance in vehicle (-) or T0901317 (T) treated BMDM. Data are mean +/− SEM (*n* = 6). ** *p* ≤ 0.01, *** *p* ≤ 0.001 in comparisons between identical LXR treatments in control versus miRNA-treated cells. (C) MiR-342-5p effects on *ABCG1* transcript abundance in vehicle (-) or T0901317 (T)-treated BMDM. Data are mean +/− SEM (*n* = 6). ** *p* ≤ 0.01, *** *p* ≤ 0.001 in comparisons between identical LXR treatments in control versus miRNA-treated cells. (D) Schematic showing proposed mechanism of miR-33 regulation and its downstream effects on ABCA1 and ABCG1, via miR-342-5p modulation of the SREBP2 autoregulatory loop.

The transcript *SREBF2* contains an intronic miRNA (miR-33) that regulates fatty acid degradation and cholesterol homeostasis [32]. In this connection, it is worth noting that type 1 and 2 IFN treatment and transfection of miR-342-5p reduced miR-33 abundance in BMDM (Fig 10A and S7C Fig). Since miR-342-5p targets the mature, spliced *SREBF2* transcript in the cytoplasm, the reduction of miR-33 must be occurring in the nucleus prior to splicing. Whilst the underlying molecular mechanism for this remains to be fully elucidated, we anticipate a decrease in miR-33 occurs as a result of miR-342-5p effects on the transcriptional autoregulation of the SREBF2 promoter by SREBP2 (Fig 10D). Since miR-33-5p targets *ABCA1* (Entrez Gene: 11303) and *ABCG1* (Entrez Gene: 11307) in mice [32], the question arose: can miR-342-5p regulate the abundance of these cholesterol efflux-related transcripts? The nuclear hormone Liver X receptors LXRα (NR1H3 UniProt: Q9Z0Y9) and LXRβ (NR1H3 UniProt: Q60644) are activated by oxysterol binding and are well-known regulators of cholesterol homeostasis acting to regulate the transcription of *ABCA1* and *ABCG1*. As a consequence, they mediate cholesterol efflux from the cell [46]. To analyse miR-342-5p modulation of *ABCA1* and *ABCG1* expression, we measured the abundance of these transcripts in transfected BMDM treated with the LXR agonist T0901312. A schematic illustrating the logic of this approach is presented (S7D Fig). MiR-342-5p suppression of miR-33 was confirmed by Q-RT-PCR in these experiments (S7E Fig) and, as previously reported, miR-33 significantly reduced the effects of LXR activation on *ABCA1* and *ABCG1* transcript abundance in our experiments (Fig 10B and 10C) [32]. In contrast, miR-342-5p enhanced the effect of T0901312 treatment on *ABCA1* and, significantly, on *ABCG1* expression (Fig 10B and 10C).

We conclude that miR-342-5p targets and acts to suppress *SREBF2* via a single, conserved miRNA binding site in its 3’UTR. In addition, our results point to a coupling of miR-342-5p to miR-33 regulation—likely through the SREBP2 transcriptional autoregulation pathway [29]. MiR-342-5p, therefore, also has the potential to counter-regulate LXR-mediated control of cholesterol homeostasis.

**Fig 11:**
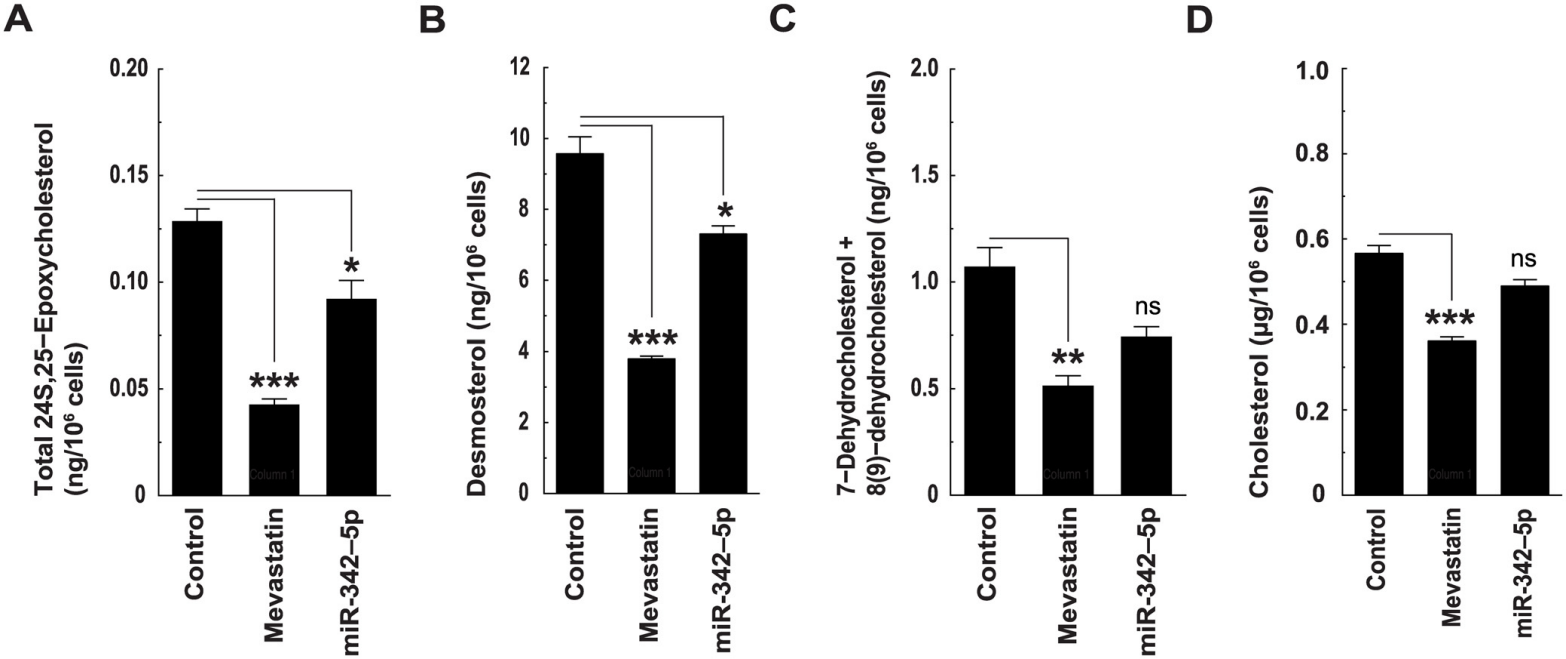
MiR-342-5p regulates intracellular cholesterol homeostasis. (A to D) Mass spectrometry quantitation of total 24S,25-Epoxycholesterol (A), Desmosterol (B), 7-Dehydrocholesterol + 8(9)-dehydrocholesterol (C) and free cholesterol (D) in BMDM transfected with miR-342-5p for 48 h. Data are mean +/− SEM (*n* = 3). * *p* ≤ 0.05, ** *p* ≤ 0.01, *** *p* ≤ 0.001.

We next investigated whether miR-342-5p could regulate intracellular sterol levels. Mass spectrometry analyses of transfected BMDM showed miR-342-5p-induced reductions in metabolites from the mevalonate-shunt (24S,25-epoxycholesterol, Fig 11A), Bloch (desmosterol, Fig 11B) and Kandutsch-Russell pathways (7-dehydrocholesterol + 8(9)-dehydrocholesterol, Fig 11C) demonstrating miR-342-5p inhibits sterol biosynthesis. Accordingly, free (Fig 11D) and total cholesterol (S8A Fig) were also reduced by miR-342-5p transfection in BMDM and fibroblasts, respectively. In agreement with our gene expression data, we further showed that an inhibitor of endogenous miR-342-5p could moderately increase total cholesterol in transfected fibroblasts (S8B Fig). In these experiments, miR-342-5p consistently reduced intracellular cholesterol concentration in cells cultured in medium containing 10% serum. This agrees with experiments showing that miR-342-5p targets biosynthesis through *SREBF2* and probably also influences other components of the cholesterol regulatory network responsible for influx and/ or efflux, e.g., ABCG1.

**Fig 12:**
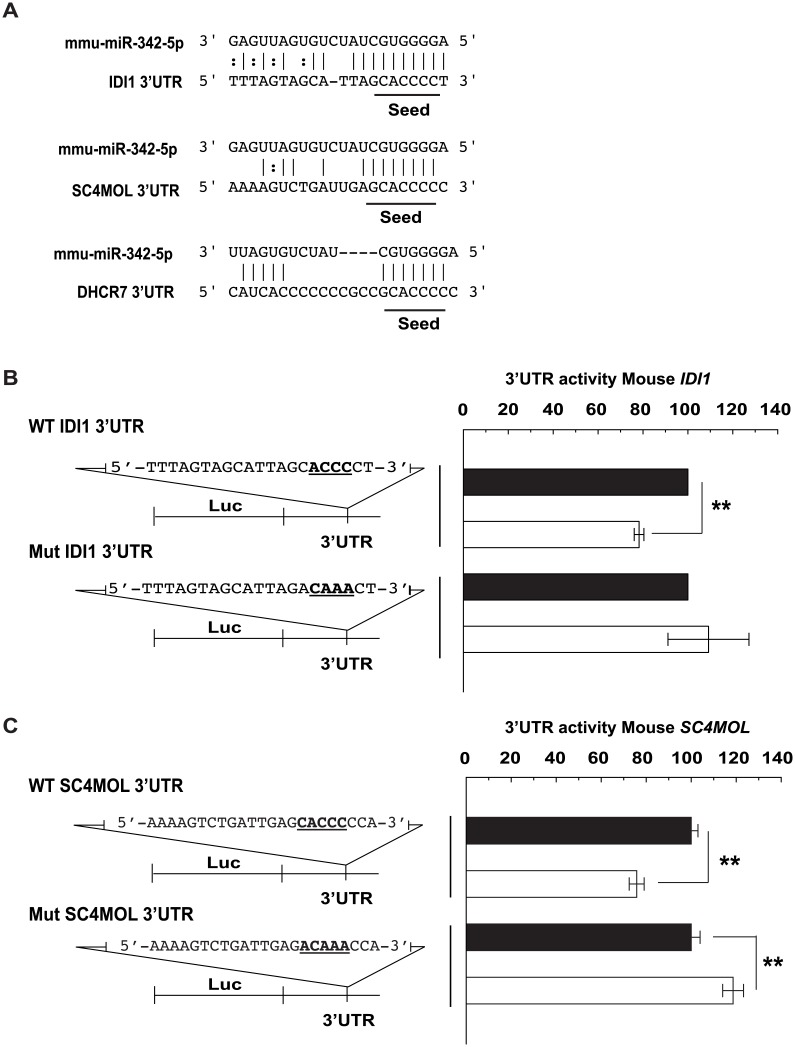
Multihit targeting of the sterol biosynthesis pathway by miR-342-5p. (A) Predicted targets for miR-342-5p in mouse *IDI1*, *SC4MOL*, and *DHCR7* transcripts. (B) Specific targeting of Mouse *IDI1* 3’UTR by miR-342-5p. Data are mean +/− SEM (*n* = 3), ** *p* ≤ 0.01. (C) Specific targeting of Mouse *SC4MOL* 3’UTR by miR-342-5p. Data are mean +/− SEM (*n* = 3), ** *p* ≤ 0.01.

Our previous studies have indicated that although IFN suppression of sterol biosynthesis and the antiviral activities of 25-HC are partly SREBP-dependent, SREBP independent sterol pathway-related mechanisms predominate in the repression of virus infection [18]. In this study, candidate targets for miR-342-5p in the sterol pathway members *IDI1* (Entrez Gene: 319554), *SC4MOL* (MSMO1 Entrez Gene: 66234), and *DHCR7* (Entrez Gene: 13360) suggested a possible SREBP-independent mechanism for miR-342-5p (Fig 12A and S1 Table). IDI1 (UniProt: P58044) in particular plays a key role in the synthesis of isoprenoids from sterol pathway metabolic intermediates—a process of great significance to a range of viruses [18,47]. Dual luciferase assays were, therefore, used to validate miRNA target sites for miR-342-5p in the 3’UTR of *IDI1* and *SC4MOL* (Fig 12B and 12C). MiR-342-5p also reduced the expression of a WT *DHCR7* 3’ UTR reporter (S8C Fig). Taken together, our data show that the IFN-induced miR-342-5p targets sterol metabolism at multiple levels in both a SREBP2-dependent and -independent manner and can regulate both cholesterol biosynthesis and homeostasis.

### MiR-342-5p Has Broad-Spectrum Antiviral Activity In Vitro and In Vivo

**Fig 13:**
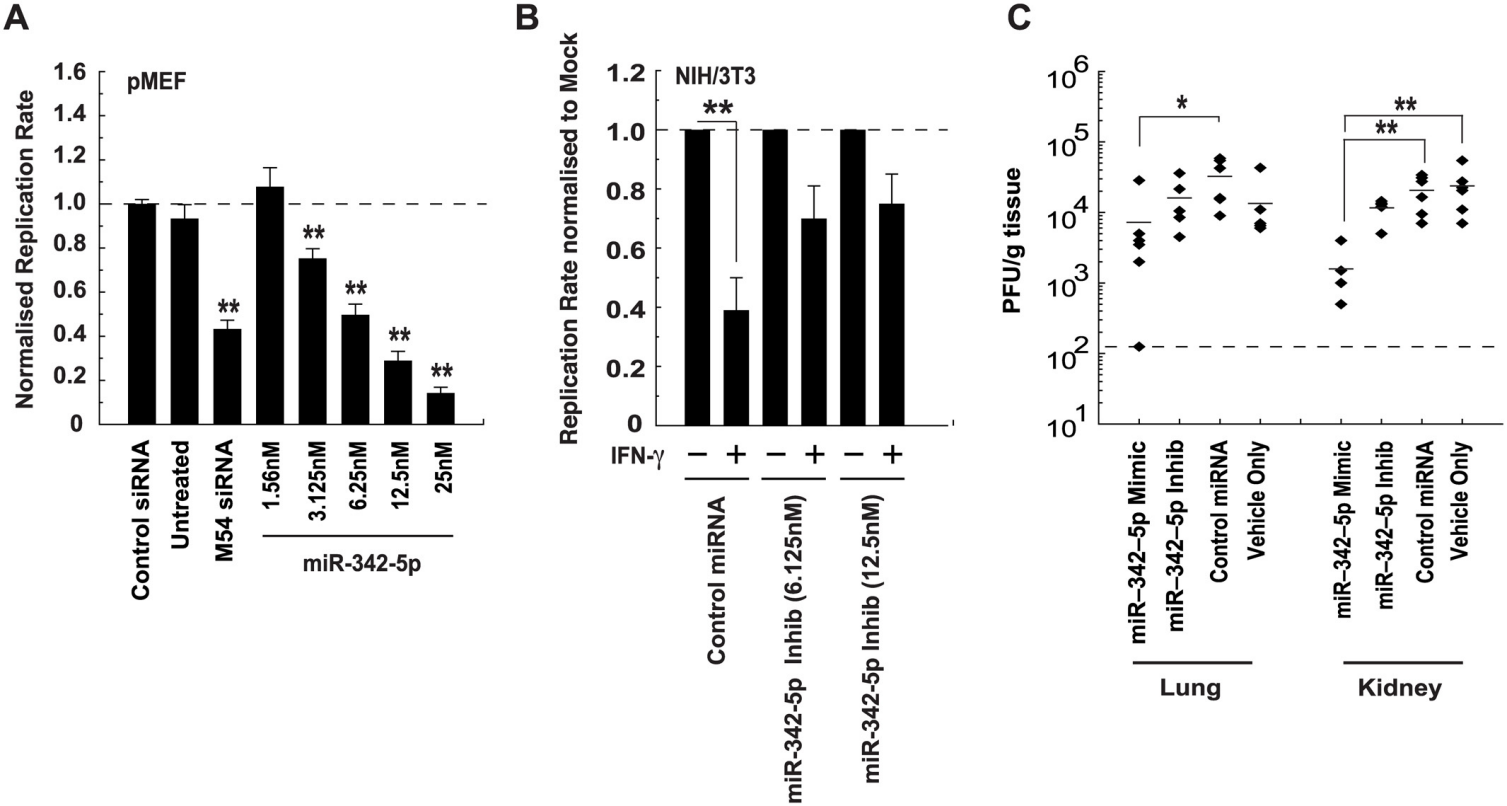
MiR-342-5p is antiviral. (A) MCMV-GFP replication is inhibited in pMEF by miR-342-5p in a dose-dependent manner. Data are normalised to values obtained with nontargeting control siRNA and are mean +/− SEM of slope between 62 and 92 hpi (*n* = 6), ** *p* < 0.01. (B) An inhibitor of miR-342-5p reduces the antiviral effect of IFN-γ. NIH/3T3 cells were transfected with 6.125 or 12.5 nM miR-342-5p inhibitor for 48 h. Cells were then mock or IFN-γ treated (100 pg/ml) for 6 h, infected with MCMV-GFP (MOI = 0.0025), and then cultured in medium containing 3% delipidised serum. Data show replication slope of virus in IFN-γ treated cells relative to Mock. Data are mean +/− SEM of slope between 62 and 92 hpi (control miRNA *n* = 4, inhibitor *n* = 6). **p ≤ 0.01. (C) MCMV titre (4 dpi) in lung and kidney of BALB/c mice treated with the miR-342-5p, miR-342-5p ZEN-AMO inhibitor or control miRNA. Data are titre in organ of individual mouse (*n* = 6). *p ≤ 0.05, **p ≤ 0.01.

We, and others, have previously demonstrated that sterol pathway regulation is an integral part of the IFN antiviral response [18,24,46]. We speculated; therefore, that the regulation of sterol biosynthesis and homeostasis by miR-342-5p might mediate antiviral functions and protect cells from virus infection. In support of this hypothesis, we found miR-342-5p but not miR-342-3p significantly inhibited growth of MCMV (S9A Fig), and that this occurred in a dose-dependent manner (Fig 13A). We further showed that an inhibitor of endogenous miR-342-5p could partially rescue MCMV replication in NIH/3T3 fibroblasts treated with IFN-γ (Fig 13B, IFN-γ function confirmed in S9B Fig). We next tested whether miR-342-5p is capable of inhibiting MCMV in vivo. BALB/c mice were injected intraperitoneally with 10 μg (total) of miR-342-5p mimic, inhibitor, or a nonspecific control RNA. Four d postinfection, MCMV titres in miR-342-5p-treated mice (relative to the nonspecific control miRNA) were significantly reduced (~1 log_10_) in the kidney and lung (Fig 13C). In further, independent in vivo experiments, antiviral miR-342-5p effects were consistently observed; however, organs with a reduced virus titre varied between runs. This is likely attributable to variability in the delivery of relatively small doses of the miRNA (S9C and S9D Fig). In our in vivo experiments, the miR-342-5p inhibitor did not increase viral replication relative to the negative control miRNA treatment (Fig 13C). While further genetic loss-of-function studies are required to unequivocally investigate the in vivo function of miR-342-5p, these studies support the view that this miRNA imparts antiviral activity in vivo and in vitro.

**Fig 14:**
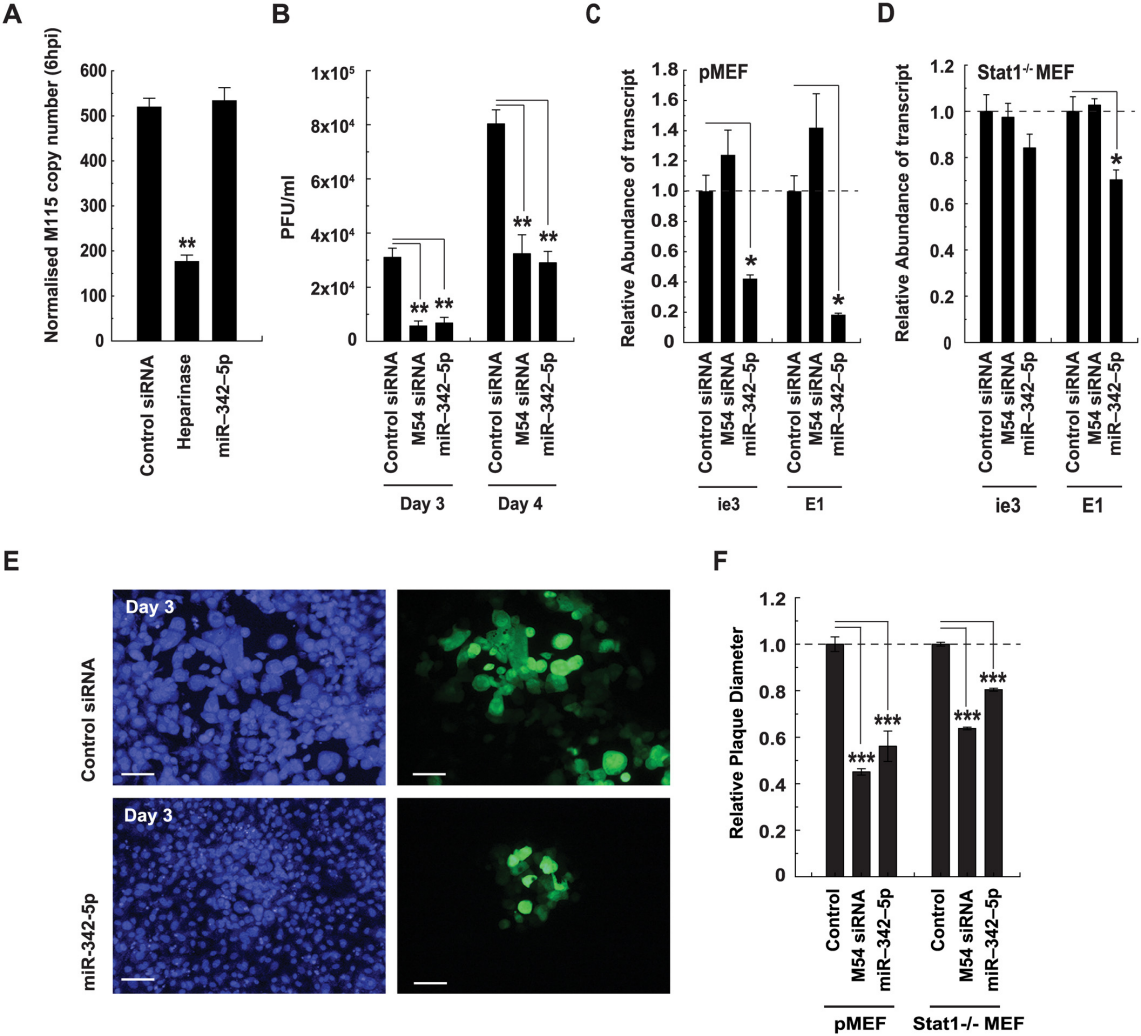
MiR-342-5p inhibits infectious virus production and spread. (A) MiR-342-5p does not affect MCMV entry. MCMV M115 DNA copy number in pMEF transfected with negative control siRNA or miR-342-5p (25 nM) for 48 h and then infected with MCMV (MOI = 0.1) for 4 h. Data are mean +/− SEM (*n* = 3). **p ≤ 0.01. (B) MiR-342-5p inhibits infectious MCMV production. PMEF were transfected (25 nM, 48 h) with nontargeting control siRNA, MCMV M54 siRNA, or miR-342-5p and then infected with MCMV (MOI = 0.0025). PFU in supernatants were then quantitated by plaque assay at 3 dpi and 4 dpi. Data are mean +/− SEM (*n* = 3). **p ≤ 0.01. (C and D) MiR-342-5p inhibits MCMV gene expression. Primary MEF (C) or STAT1^-/-^ MEF (D) were transfected (25 nM, 48 h) with nontargeting control siRNA, MCMV M54 siRNA, or miR-342-5p and then infected with MCMV (MOI = 0.01). MCMV transcript abundance was then quantitated by Q-RT-PCR at 6 hpi. Data are mean +/− SEM (WT, *n* = 3, STAT1^-/-^ *n* = 4). *p ≤ 0.05. (E) Images showing effects of miR-342-5p on MCMV-GFP infected cells at 3 dpi. Bar = 100 μm. (F) MiR-342-5p effects on plaque formation were analysed at 4 dpi when monolayers were fixed and plaque diameter quantitated using ImageJ software. Data are mean +/− SEM, *n* = 4. For pMEF: Control = 168 plaques counted, M54s siRNA 66 plaques counted, miR-342-5p *n* = 23. STAT1-/- MEF: Control = 1,091 plaques counted, M54 siRNA = 822 plaques counted, miR-342-5p = 1,011 plaques counted. ***p ≤ 0.001

**Fig 15:**
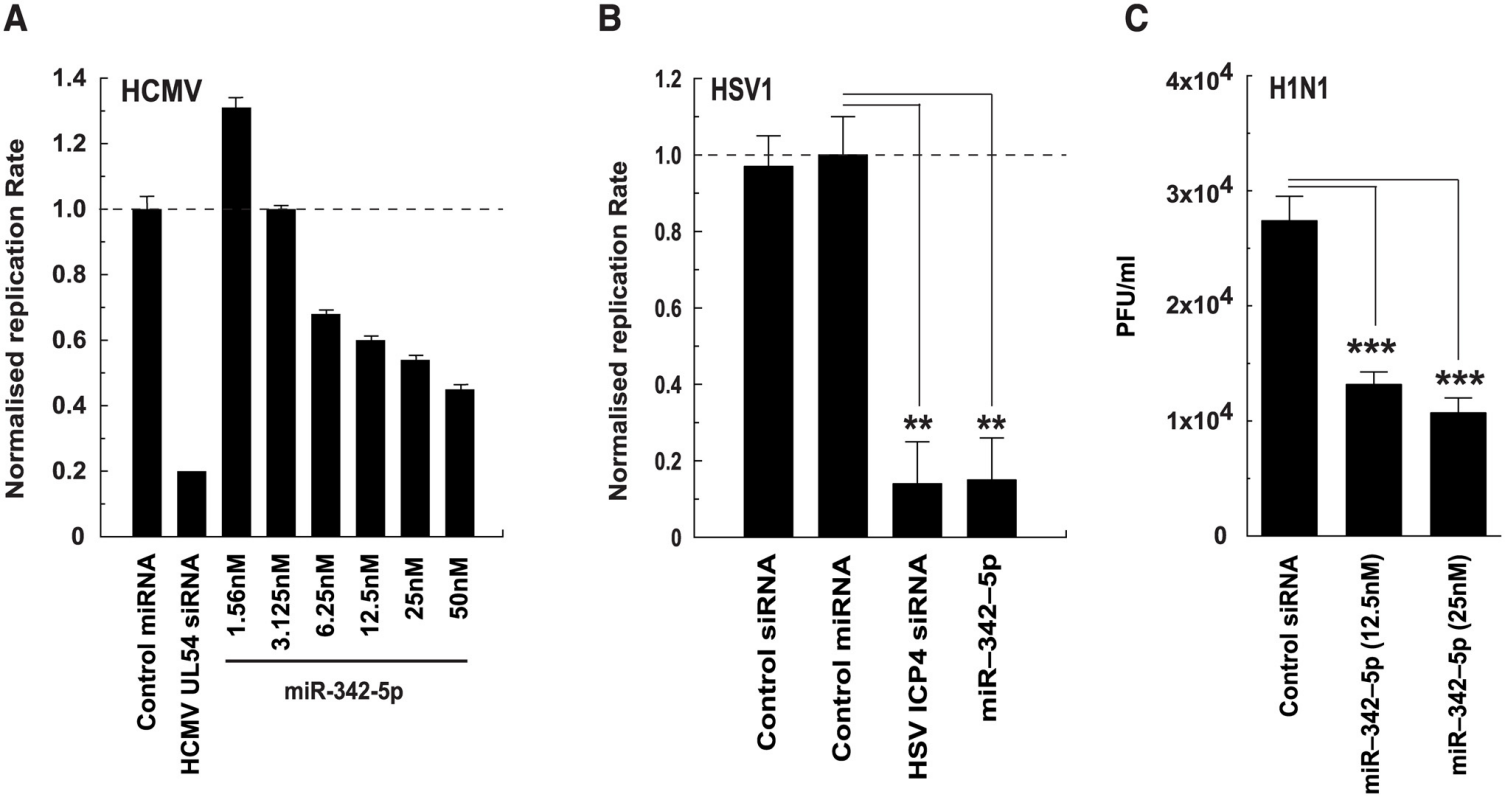
MiR-342-5p exerts a broad antiviral activity. (A) Inhibition of HCMV-GFP in MRC5 cells. Data are normalised to values obtained with *C*. *elegans* miRNA and are mean +/− SEM (*n* = 2). (B) Inhibition of HSV1-GFP in HeLa cells. Data are normalised to values obtained with *C*. *elegans* control miRNA and are mean +/− SEM (*n* = 3), ** *p* ≤ 0.01. (C) Inhibition of Influenza virus (H1N1). A549 cells were transfected with miRNA for 48 h then infected with H1N1 for 24 h. Infectious virus titre in supernatants was then analysed by plaque assay. Data are mean +/− SEM. Control siRNA and miR-342-5p (25 nM) *n* = 14. MiR-342-5p (12.5 nM) *n* = 6. *** *p* ≤ 0.001.

We have previously reported that IFN-induced effects arising via sterol pathway regulation play a key role in limiting MCMV replication and are mediated via the isoprenoid-prenylation branch [18]. This results in severely restricted viral spread at a postentry stage of infection. In agreement with our 25-HC-related work, miR-342-5p transfection of cells did not alter viral entry (Fig 14A) but did significantly reduce infectious virus production (Fig 14B), immediate early gene expression (Fig 14C and 14D), and MCMV plaque diameter. Fig 14E shows a miR-342-5p elicited reduction in the diameter of plaques at 3 d post infection, whilst Fig 14F shows data from a quantitative analysis of plaque diameter in both WT and STAT1^-/-^ cells at 4dpi. The latter cell type was used to minimise potential side effects of transfection on immune activation and subsequent antiviral responses. Of note, miR-342-5p did not notably alter the viability of cells used in any of the described analyses (S10A and S10B Fig). Since miR-342-5p targets the sterol metabolic network, we tested whether it exerted broader antiviral activity by analysing its effects on Human Cytomegalovirus (HCMV), Herpes Simplex virus 1 (HSV1), and Influenza A virus (H1N1) (Fig 15A, 15B and 15C). Replication of all three viruses was inhibited by miR-342-5p (80% for HSV-1, 50% for HCMV and 60% for Influenza virus A).

### Broad Differential Antiviral Effects of miR-342-5p Are Mediated through Multihit Targeting of the Sterol Pathway

**Fig 16:**
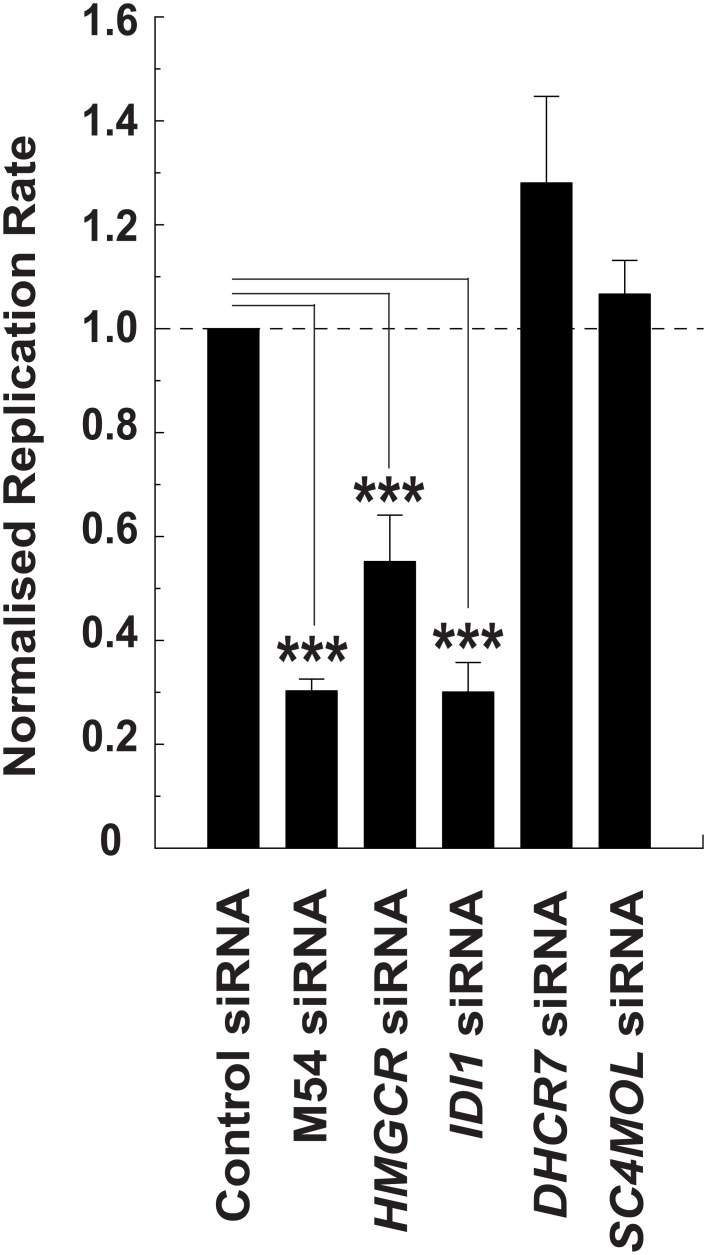
Knockdown of *IDI1* but not *DHCR7* or *SC4MOL* results in inhibition of MCMV replication in pMEF. MCMV-GFP replication in pMEF transfected with *HMGCR*, *IDI1*, *DHCR7* or *SC4MOL*-specific siRNA. Data are mean +/- SEM, (*n* = 3) *** *p* ≤ 0.001.

As previously discussed, whilst IFN-induced suppression of sterol biosynthesis involves a SREBP-dependent mode of action, alternative sterol pathway-related mechanisms predominate in the repression of virus replication [18]. With this in mind, we sought to further investigate SREBP-independent antiviral mechanisms for miR-342-5p. For these experiments, we evaluated whether the specific knockdown of predicted non-SREBP targets of miR-342-5p (*IDI1*, *DHCR7* and *SC4MOL*) alone were sufficient to impart antiviral effects observed for the miRNA (Fig 16). Knockdown of specific targets by these siRNA was confirmed in pMEF by Q-RT-PCR (S9F Fig). Notably, siRNA-mediated knockdown of *IDI1* consistently inhibited (−70%) MCMV replication in pMEF with a similar magnitude as inhibition mediated by a virus-specific (M54, MCMV DNA polymerase) or *HMGCR*-targeting siRNA (Fig 16). In contrast, siRNA targeting of *SC4MOL* and *DHCR7*—enzymes that function downstream of the isoprenoid pathway branch—did not limit MCMV replication. Fig 12C shows functional targeting of *SC4MOL*. Since SC4MOL catalyses six distinct enzymatic steps in the Kandutsch-Russell/Bloch arms of the sterol biosynthesis pathway (Fig 4), we anticipate miR-342-5p targeting of this enzyme could have significant effects on cholesterol biosynthesis in the cell and antiviral effects on pathogens dependent on this arm of the pathway. This data is in agreement with previous studies and demonstrates that SREBP-independent targeting of the sterol biosynthesis pathway can elicit a profound antiviral effect [18,27].

**Fig 17:**
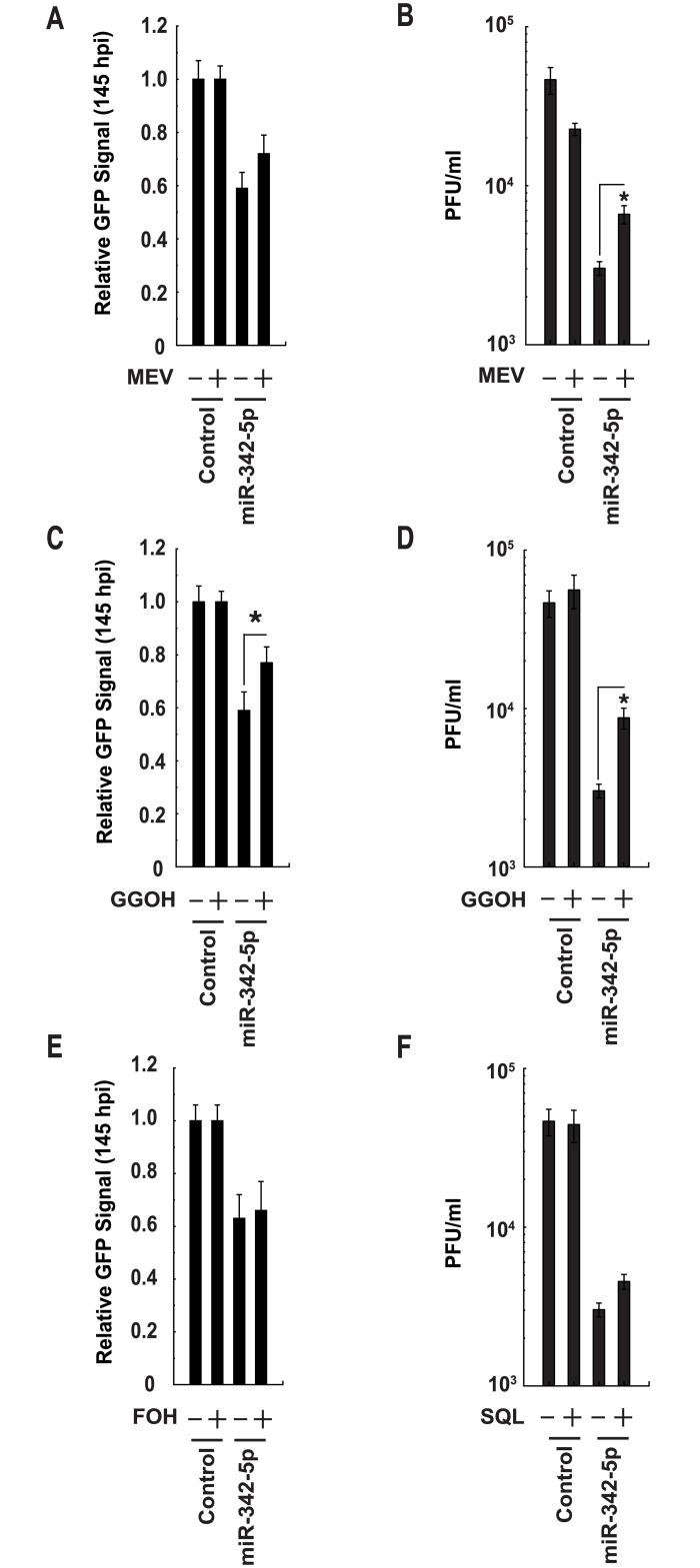
Inhibitory effects of miR-342-5p on MCMV are mediated through regulation of the mevalonate-isoprenoid pathway. (A) MCMV-GFP replication in pMEF transfected with control miRNA or miR-342-5p and then cultured with MEV (1 μM final concentration). Data are mean +/− SEM (*n* = 39). (B) MCMV PFU production in pMEF transfected with control miRNA or miR-342-5p and then cultured with MEV (20 μM final concentration). Data are mean +/− SEM (*n* = 4). * *p* ≤ 0.05. (C) MCMV-GFP replication in pMEF transfected with control miRNA or miR-342-5p and then cultured with GGOH (1 μM final concentration). Data are mean +/− SEM (*n* = 39). * *p* ≤ 0.05. (D) MCMV PFU production in pMEF transfected with control miRNA or miR-342-5p and then cultured with GGOH (20 μM final concentration). Data are mean +/− SEM (*n* = 4). * *p* ≤ 0.05. (E) MCMV-GFP replication in pMEF transfected with control miRNA or miR-342-5p and then cultured with FOH (1 μM final concentration). Data are mean +/− SEM (*n* = 19). (F) MCMV PFU production in pMEF transfected with control miRNA or miR-342-5p and then cultured with SQL (20 μM final concentration). Data are mean +/− SEM (*n* = 3). * *p* ≤ 0.05.

It is known that unrelated viruses have a common dependency on the mevalonate- sterol pathway. Evidence suggests, however, that the relative significance of individual pathway members differs according to virus type and the respective demands of its replication strategy [48,49]. In the case of MCMV, we have previously demonstrated that the addition of exogenous metabolic intermediates (mevalonate or geranylgeranyl diphosphate but not squalene [SQL]) to cells can partially rescue MCMV growth in vitro by compensating for IFN or 25-HC-induced suppression of the sterol pathway [18,24]. In this study, we sought to test whether the antiviral effects of miR-342-5p require the suppression of metabolic intermediates in the mevalonate and sterol biosynthesis arms of the pathway. In a series of MCMV metabolite rescue experiments, and in agreement with our 25-HC-related work, we found that geranylgeraniol (GGOH) (Fig 17C and 17D) and, to a lesser degree, mevalanolactone (MEV) (Fig 17A and 17B) (but not farnesol [FOH] or SQL, Fig 17E and 17F), could significantly increase and partially restore MCMV growth in miR-342-5p transfected pMEF. These results highlight the importance of host-targeting of the mevalonate arm of the sterol pathway by miR-342-5p and are in good agreement with our previous studies [18,24].

**Fig 18:**
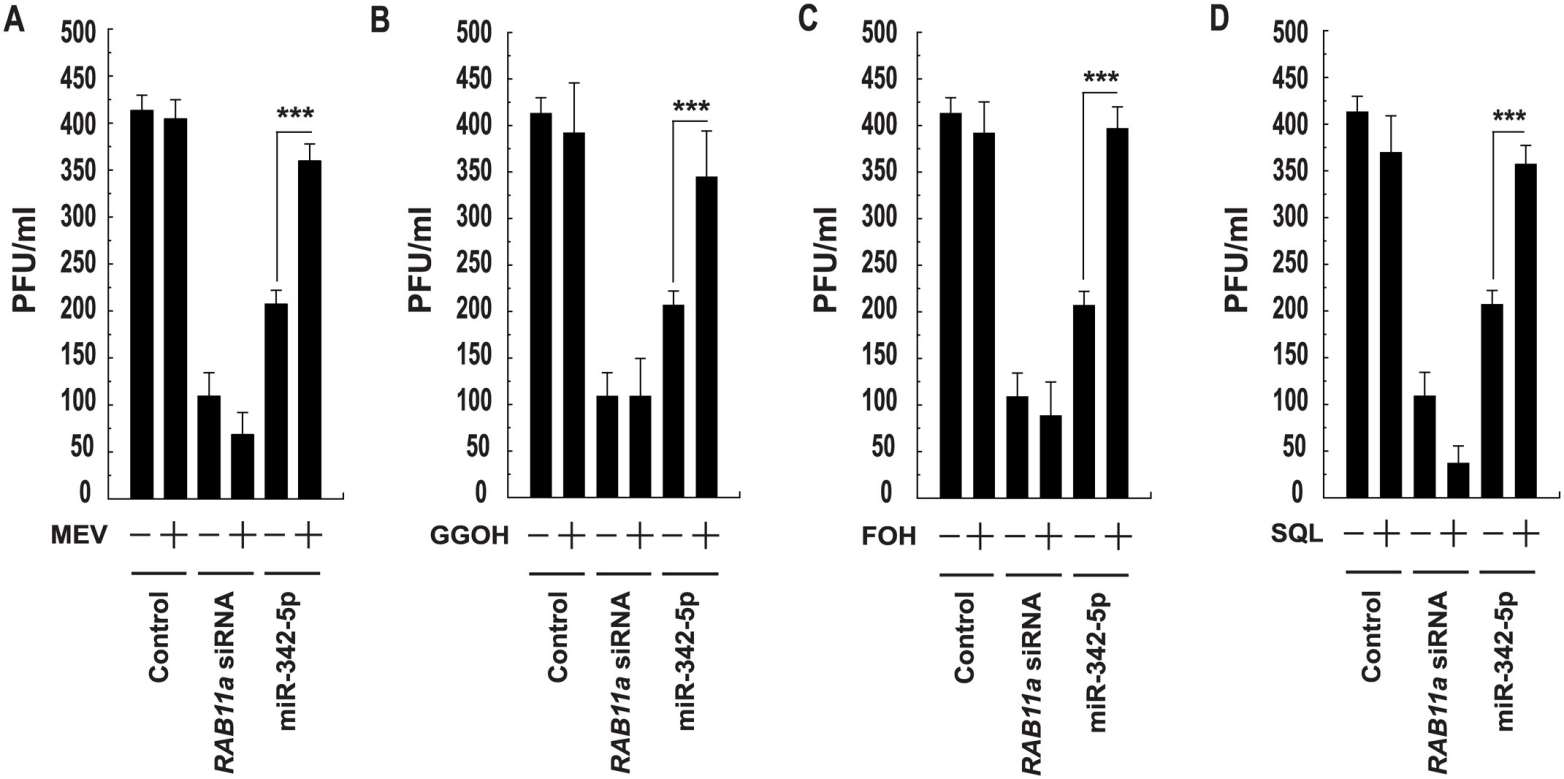
Effects of miR-342-5p on Influenza virus are mediated through regulation of the mevalonate and sterol arms of the biosynthesis pathway. (A to D) Influenza virus A (H1N1) PFU production in A549 cells transfected with control miRNA or miR-342-5p for 48 h and then infected with H1N1 (MOI = 0.1) and cultured with MEV (A, 10 μM, *n* = 8), GGOH (B, 10 μM, *n* = 12), FOH (C, 10 μM, *n* = 12) or SQL (D, 10 μM, *n* = 12) for 24 h.

In contrast to MCMV, a series of Influenza virus metabolite rescue experiments demonstrated that MEV, FOH, SQL and, to a lesser extent, GGOH all partially rescued infectious virus production by A549 cells in the context of miR-342-5p but not RAB11A siRNA transfection (Fig 18A, 18B, 18C and 18D). RAB11 (UniProt: P62491) is known to be required for Influenza A virus budding and filament formation [50]. Whilst caution should be taken to avoid overinterpreting the relative effects of specific metabolites in unrelated cell types, when taken together, our metabolite rescue data emphasise two key mechanistic issues. Firstly, unrelated viruses depend on distinct metabolic aspects of the sterol biosynthesis pathway for their replication. Secondly, despite these distinctions, “multihit” targeting of the sterol pathway by miR-342-5p enables this miRNA to elicit a broad antiviral effect.

MiR-342-5p has recently been reported to specifically target Cocksackie B virus [51]. While we cannot exclude the possibility that miR-342-5p also directly targets MCMV, HCMV, and/or HSV-1 (S2 Table), we failed to find any direct viral-RNA targets for the miRNA in Influenza virus A. Given the relatively low number of cellular miRNAs shown to have antiviral activity [5], we consider it unlikely that miR-342-5p exerts broad antiviral effects by directly targeting viral transcripts. On the basis of these observations, combined with evidence from the metabolic rescue experiments, we propose that miR-342-5p exerts its broad antiviral effects by targeting host metabolic activities associated with the mevalonate-sterol biosynthesis pathway.

## Discussion

In this study, we identify miR-342-5p as a cellular miRNA with broad antiviral properties whose transcriptional regulation is coupled to IFN signalling by IRF1. MiR-342-5p is, therefore, an integral component of the cell-autonomous IFN response and exerts its antiviral effects by inhibiting the sterol metabolic network through SREBP-dependent and -independent mechanisms. The latter are, at least in part, mediated via the targeting of key genes in the sterol metabolic network. Metabolic rescue experiments show that unrelated viruses have a common requirement for the mevalonate biosynthesis arm yet depend on subtly different aspects of the distal sterol pathway for their life cycle.

In its role, miR-342-5p complements and reinforces the antiviral functions of the rapidly induced oxysterol 25-HC on sterol biosynthesis—in particular affecting the mevalonate-isoprenoid branch of the pathway. S11 Fig summarises our current understanding of the mechanisms by which IFN induced 25-HC and miR-342-5p function to coordinately regulate sterol biosynthesis. In murine BMDM, synthesis of *CH25H* mRNA is controlled by STAT1 and is up-regulated in the first 30 min after cells are activated by IFN-γ [18]. In contrast, *EVL* and pri/pre-miR-342 RNA expression increases 2 h to 3 h after IFN-γ activation of BMDM and is regulated by IRF1. Our data demonstrate, therefore, an IFN-elicited sequential regulation of the sterol metabolic network in which 25-HC provides an immediate, rapid mechanism for decreasing sterol biosynthesis and mediating antiviral effects. This involves a 25-HC blockade of SREBP2 translocation to the nucleus and the proteolytic degradation of HMGCR [18,52]. The effects of 25-HC are followed by miR-342-5p further promoting a more sustained fine-tuning of sterol metabolism and antiviral effects in the cell by targeting *SREBF2* RNA and transcripts encoding select enzymes of the sterol biosynthesis pathway (e.g., *IDI1* and *SC4MOL*).

25-HC has recently been shown to mediate anti-inflammatory activities via SREBP2 [30]. Here, we find miR-342-5p functions in a SREBP-dependent and -independent manner to regulate sterol biosynthesis and viral infection. Whether miR-342-5p has SREBP-related inflammatory functions remains to be fully investigated; however, the current study strongly supports the possibility that miR-342-5p might contribute to the regulation of inflammatory responses by targeting SREBP2 or the prenylation arm of the sterol pathway. In this context, a proinflammatory role for miR-342-5p in the enhancement of miR-155 expression has been described and attributed to the former miRNA targeting *BMPR1* (Entrez Gene: 12166) and *AKT1* (Entrez Gene: 11651) transcripts in macrophages [42]. In this study, we did not observe any notable alterations in *BMPR1* transcript synthesis or abundance during 8 h of IFN-γ BMDM treatment. We did, however, observe a small but significant reduction in *AKT1* abundance over time in IFN-stimulated macrophages. A knockdown of this transcript with a siRNA, however, had no effect on MCMV replication (S9E Fig).

Intriguingly, miR-342-5p targets multiple members of the sterol biosynthesis pathway including its master regulator SREBP2. The suppression of SREBP2 function results in a generalised decrease in sterol biosynthesis as well as a reduction in the abundance of key contributors to cholesterol homeostasis such as *INSIG1* (Entrez Gene: 231070; Fig 7C, S2 and S3 Figs) and *LDLR* (Entrez Gene: 16835; S8D Fig). Both DNA and RNA viruses require the sterol biosynthesis pathway for optimal growth capacity, and we have recently shown that an IFN-mediated suppression of sterol metabolic network activity is an integral part of the antiviral response [24]. Importantly, however, whilst the IFN regulation of SREBP2 is undoubtedly important, alternative mechanisms play a dominant role in the sterol-related antiviral responses we have observed to-date.

Notably, in this study we found miR-342-5p directly targets enzymes of both the proximal and distal arms of the sterol biosynthesis pathway—in particular IDI1. IDI1 is an enzyme critical to protein prenylation and catalyses the isomerisation of the inactive carbon-carbon double bond of isopentyl diphosphate (IPP) to generate an isomer dimethylallyl diphosphate (DMAPP). Prenylation is a post-translational modification enabling the membrane association of modified proteins and involves the covalent addition of prenyl lipids (e.g., farnesyl or geranylgeranyl) derived from mevalonic acid to conserved residues at the C-terminus of proteins. This process is integral to host intracellular protein trafficking, leukocyte chemotaxis, and phagocytosis and has also been implicated in inflammatory cytokine production [53–56]. In this connection, we have recently highlighted the importance of the mevalonate-isoprenoid branch point to the antiviral effects of 25-HC [24].

The replication of several viruses requires prenylation of host and/or virus proteins. For example, Hepatitis D virus requires prenylation of its large delta antigen for optimal virion morphogenesis, and prenylation inhibitors have shown promise in the treatment of this pathogen [48]. Further, Hepatitis C virus requires the geranylgeranylated host protein FBL2 for replication and respiratory syncytial virus (RSV) F glycoprotein binds to the prenylated host protein RHOA enabling membrane fusion [57,58]. Here, we found that siRNA knockdown of IDI1 inhibits MCMV replication in pMEF [18] and, most importantly, the inhibitory effects of miR-342-5p on MCMV could be partially rescued via exogenous administration of GGOH and, to a lesser degree, MVA to cells. This points to an involvement of the sterol pathway prenylation branch point in the anti-MCMV functionality of miR-342-5p. However, the mode of action of miR-342-5p on the prenylation branch point remains to be determined. For Influenza virus, the inhibitory effects of miR-342-5p were partially rescued via the administration of exogenous MEV, FOH, SQL and, to a lesser extent, GGOH to cells. Thus, different targeting mechanisms of miR-342-5p facilitate broad antiviral effector functions.

The metabolic rescue data described above is crucial to our findings. It confirms sterol pathway targeting is essential to the antiviral effects of miR-342-5p we observed and highlights that whilst many viruses depend on the sterol metabolic network for their replication, unrelated virus types may exploit subtly different aspects of this pathway for their replication cycle. As a result, “multihit” targeting of a single pathway by miR-342-5p has beneficial outcomes for its broad antiviral effects.

Studies supporting a role for antiviral RNA-mediated interference in mammalian systems are beginning to emerge; however, the IFN response is still considered the pre-eminent mechanism by which mammalian cells resist viral infection [59–61]. Specific targeting of viral transcripts by host miRNAs has been reported, e.g., for hepatitis C virus (HCV) [6]. However, this is unlikely to represent a major mechanism for restricting viruses, as they are predisposed to the emergence of escape mutants [62]. In contrast, IFN-elicited miRNAs targeting cellular pathways required for virus replication arguably provide more robust and durable effects against a broad spectrum of viruses. Here, to our knowledge, we identify for the first time an IFN-induced host-targeting miRNA that elicits broad antiviral effects. Whilst we cannot preclude the possibility that miR-342-5p has a direct effect on viral RNA expression, the breadth of viruses repressed by this miRNA (both DNA and RNA) argues against a direct targeting of viral transcripts. Although miR-342-5p has been shown to specifically inhibit coxsackie B virus [51], our work emphasises the multifunctional, dual potential of this IFN-regulated antiviral miRNA.

In conclusion, the multihit targeting of sterol synthesis and antiviral effects mediated by miR-342-5p represent a new arm of the IFN-induced cell-autonomous immune response to viral infection and a new mechanistic link between lipid metabolism and the very early innate immune response. In this regard, miR-342-5p and its sterol pathway targets provide the foundation for future therapeutic exploitation, and this study highlights a general principle for blockade of infection.

## Materials and Methods

### Mice

C57BL/6 mice were housed in the specific pathogen-free animal facility at the University of Edinburgh. BALB/c mice were housed in the specific pathogen-free animal facility at the Institut d’Investigacions Biomèdiques August Pi i Sunyer, Barcelona, Spain. CH25H^-/-^ (B6.129S6-Ch25h^tm1Rus^/J) mice were purchased from Charles River (Margate, United Kingdom) and housed in the specific pathogen-free animal facility at the University of Edinburgh. All procedures were carried out under project and personal licences approved by the Secretary of State for the Home Office, under the United Kingdom's 1986 Animals (Scientific Procedures) Act and the Local Ethical Review Committee at Edinburgh University. All procedures involving animals and their care in Spain were approved by the Ethics Committee (protocol number CEEA 308/12) of the University of Barcelona and were conducted in compliance with institutional guidelines as well as with national (Generalitat de Catalunya decree 214/1997, DOGC 2450) and international (Guide for the Care and Use of Laboratory Animals, National Institutes of Health, 85–23, 1985) laws and policies.

### Cell Propagation and Culture

BMDMs were isolated and differentiated with CSF-1 derived from L929 cells. Details of all cell culture conditions are provided in S1 Methods.

### Treatment of Cells with IFN or LPS

Unless otherwise stated, murine recombinant IFN gamma (IFN-γ) (Perbio Science) and IFN-β (Stratech, UK) were added to cells at a final concentration of 10 U/ml or 25 U/ml, respectively. For experiments investigating the effect of the miR-342-5p inhibitor on SREBF2 transcript abundance in BMDM, subconfluent macrophages were transfected for 24 h and then treated with 2.5 ng/ml murine recombinant IFNG (Life Technologies) for 24 h. Lipopolysaccharides from *Escherichia coli* 026:B6 (Sigma-Aldrich, UK) were reconstituted in SPBS (1 mg/ml) and added to BMDM at a final concentration of 100 ng/ml.

### Reporter Viruses and Viral Plaque Assay

The GFP-encoding MCMV (MCMV-GFP) has been previously described [63]. HCMV-GFP (AD169-GFP) has been previously described [64]. HSV-1-eGFP (C12) was propagated and titred by plaque assay in Vero cells. A/WSN/33 (H1N1) influenza virus was propagated and titred in MDCK cells. Growth and titration conditions are provided in S1 Methods.

### GFP-Virus growth

MiRNA mimics or siRNA were transfected into cells using DharmaFECT 1 (Thermo Fisher Scientific). After 48 h or 72 h, cells were infected with virus and growth was measured using a POLARstar OPTIMA plate reader (BMG Labtech) according to manufacturers’ recommendations. See S1 Methods.

### BMDM IFN-γ Treatment, RNA Labelling and Isolation

Incorporation of 4-thiouridine (Sigma) into newly-transcribed RNA was undertaken as described by Dölken et al. [65]. See S1 Methods for further information.

### Newly Transcribed RNA Labelling for Microarray Analysis

Processing of ntRNA samples (100 ng) for hybridisation to Affymetrix Mouse Gene 1.0 ST arrays was undertaken according to manufacturer’s instructions (Affymetrix). Hybridisation, washing, staining, and scanning of the arrays were also undertaken following standard Affymetrix protocols. After scanning and data capture, open-source R-based software “Bioconductor” was used to implement all quality control and statistical analyses. See S1 Methods for further information.

### Q-RT-PCR Analysis of Cholesterol Pathway Genes

Total RNA was extracted from cells with RNeasy Mini kit (QIAGEN). Quantitative gene-expression analyses were then performed using Roche UPL reagents, IDT PrimeTime (IDT, United States) assays or Taqman primer probe sets (Applied Biosystems). Expression of target genes was normalized to Actb unless otherwise stated. See S1 Methods for further detailed information.

### PCR Analysis of Mature miRNA

Total RNA from in vitro tissue culture experiments was isolated using a Qiagen miRNeasy kit according to manufacturer’s recommendations (Qiagen, US). MiRNA expression analyses were then performed using reagents and assays from Quanta Biosciences, US as per manufacturers recommendations. See S1 Methods for further detailed information.

### PCR Data Analysis

Stratagene MXPro software was used to analyse the data. Threshold determinations and differences in transcript abundance relative to controls were automatically performed by software for each reaction.

### Transfection of Mimics, Inhibitors, and siRNA

MicroRNA mimics, control miRNAs and siRNA were purchased from Dharmacon RNAi Technologies, Thermo Fisher Scientific (Lafayette, USA). Mimics and siRNA were transfected into cells using DharmaFECT 1 (Thermo Fisher Scientific). ZEN-AMO and 2’OMe/LNA-PS miRNA inhibitors were obtained from IDT and for NIH/3T3 cells were transfected using DharmaFECT 1 [66]. For BMDM or RAW cells, miRNA mimics or inhibitors were transfected (final concentration 25 nM unless otherwise stated) into cells using Viromer Blue (Lipocalyx, Germany) as per manufacturers recommendations. See S1 Methods for further information.

### Administration of Small RNAs to Mice

For in vivo experiments, miRNA were administered by an intraperitoneal injection route as previously described [67]. For infection, mice were injected with 1 x 10^6^ PFU MCMV in SPBS on day 3 of the experiment. Mock-infected animals were injected in an identical manner with SPBS only. Tissues were collected and snap frozen for subsequent analysis of MCMV titre. See S1 Methods for further information.

### Transfection of 3’UTR Luciferase Reporter Plasmids and miRNA

Regions from the 3’UTR of the gene were synthesized and subcloned into the 3’UTR MCS of the psiCheck2 renilla luciferase by Eurofins MWG Operon (Ebersberg, Germany). MiRNA mimics or controls were reverse transfected into cells with either wild-type or mutant luciferase reporter in DharmaFECT DUO. After 24 h, luciferase expression in the transfected cells was measured using a dual-luciferase reporter assay kit (Promega, UK). See S1 Methods for further information.

### Treatment of Cells with 25-HC or (2-Hydroxypropyl)-β-Cyclodextrin

25-HC (Sigma, H1015) was dissolved in 100% Ethanol (1000x stock, 20 mM) and stored at −20°C under argon in 2 ml opaque tubes with gasket screw-top lids. The powder (2-Hydroxypropyl)-β-cyclodextrin (HβCD) (Sigma, H107) was dissolved in medium at 37°C just before use.

### Measurement of Total Cholesterol Concentration by Enzymatic Assay

Cholesterol was extracted from cells using Chloroform/Methanol protocol detailed in S1 Methods. Total Cholesterol quantitation was carried out using an Amplex Red Cholesterol Assay Kit as per manufacturers recommendations (Invitrogen, UK).

### Analysis of Sterols by Mass Spectrometry

Sterols and oxysterols were analysed using liquid chromatography—mass spectrometry (LC-MS) on an Orbitrap Elite (ThermoFisher) operated as described previously[68]. Sterols and oxysterols were identified by comparison of *m/z*, retention time and MS^n^ fragmentation with reference standards. Quantification was by stable isotope-dilution. See S1 Methods for further information.

### Computational Prediction of miRNA Target Binding Sites in Sterol-Related Genes

For the prediction of potential microRNA targets in genomic 3’ UTR regions the database TargetScan was used [69]. For the prediction of microRNA targets in 5’UTR, coding regions and 3’UTRs the database miRWalk was used [70].

### Computational Prediction of miRNA Target Binding Sites in Virus Genes

MiRanda version 3.0 was used to scan viral coding sequences for predicted miRNA binding sites. The viral coding sequences were obtained from GenBank via NCBI for the following accessions: MCMV (NC_004065.1), HCMV (FJ527563.1), HSV1 (X14112.1), and influenza A (A/WSN/33 (H1N1): X14112.1, CY010795.1, CY010794.1, CY010793.1, CY010788.1, CY010791.1, CY010790.1, CY010789.1 and CY010792.1). MiRanda results were extracted as key-value pairs and sorted according to total score and free energy. See S1 Methods for further information.

### Computational Prediction of Transcription Factor Binding Sites in Evl Promoter from Human and Mouse

To analyse and predict potential Stat1, Irf1 and Irf9 binding sites in the promoter of Human and Mouse EVL/Evl, the open source software Toucan was used [71]. See S1 Methods for further information.

### Chromatin Immunoprecipitation and PCR Analysis of Promoter Sequence Enrichment

BMDM were treated with Ifn-γ (10 U/ml) for 2, 6, and 24 h, fixed and then Chromatin immunoprecipitation (ChIP) was performed as described previously [72]. Primers for the amplification of promoter regions from EVL and the positive control gene CXCL10 were designed using PrimerBLAST and are provided in S5 Fig. Quantitative-PCR using SYBR-green incorporation (Quanta PerfeCTa SYBR Green FastMix, Low ROX) was used to analyse enrichment of sequences relative to input DNA. See S1 Methods for detailed information.

### Analysis of EVL Promoter Activation

A 421-bp region from the Human EVL promoter containing three predicted IFN-activated transcription factor binding sites was synthesized and subcloned into the MCS of the pGL4.1 luciferase plasmid by Eurofins MWG Operon (Ebersberg, Germany). In parallel, corresponding mutants of each individual site (designated: ISRE, proximal Irf7 and distal Irf7) and a mutant in which all predicted sites were mutated (designated: All) were produced. Promoter activation by type 1 IFN was then tested as described in S1 Methods.

### Metabolite and LXR Agonist Treatment of Cells

After washing, normal medium containing vehicle (Ethanol) or GGOH (Sigma G3278), Mevalonolactone (MEV) (Sigma M4667), FOH (Sigma F203), or SQL (Sigma S3626) was added to the infected wells. T0901317 (Tocris Bioscience, Bristol, UK) was resuspended in 100% ethanol (5 mM stock), diluted in normal medium (50 nM final concentration) and cells were treated for 18 h.

### Statistical Analyses

Unless otherwise stated, a two-sample Welch *t* test was used to test statistical significance of results in Microsoft Excel. Prior to parametric testing, a Shapiro-Wilk test was undertaken in R to confirm normal distribution of data. Statistical testing of Q-RT-PCR data from independent experiments (normalised to housekeeping gene ACTB) was undertaken using a one-sample *t* test in Microsoft Excel. Experimental group sizes (where *n* = number of independent biological samples per group) are stated in figure legends. Statistical analyses of growth curves was undertaken using a permutation based approach using a browser-based implementation of the “compareGrowthCurves” function originally developed for the statmod software package for R (http://bioinf.wehi.edu.au/software/compareCurves).

### Cholesterol Biosynthesis Model

The cholesterol biosynthesis model used in this study was derived from a comprehensive consensus Systems Biology Graphical Notation diagram of the regulation and feedback of cholesterol metabolism [28].

## Supporting Information

S1 Data Numerical values underlying summary data displayed in the figures. (XLSX)

S1 Fig Labelling and isolation of newly transcribed RNA. (EPS)

S2 Fig Ifng down-regulates synthesis of sterol pathway transcripts. Upper Graphs: Sequential analysis of sterol pathway-related transcript synthesis (every 30 min) in IFN-γ-treated BMDM (relative to mock). Log_2_ fold change values were calculated by subtracting the Mock from the IFN-γ-treated Log_2_ scale signal value. Lower graphs: Sequential analysis of *SREBF1*, *SREBF2*, *INSIG1*, *INSIG2*, and *SCAP* transcript synthesis (every 30 min) in IFN-γ-treated BMDM (relative to mock). Log_2_ fold change values were calculated as described for (B).
(EPS)

S3 Fig Ifng down-regulates abundance of sterol pathway transcripts. Upper graphs: Abundance of sterol biosynthesis pathway-related transcripts in IFN-γ-treated BMDM (relative to mock). Log_2_ fold change values were calculated by subtracting the Mock from the IFN-γ treated Log_2_ scale signal value. Lower graphs: Abundance of *SREBF1*, *SREBF2*, *INSIG1*, *INSIG2*, and *SCAP* transcripts in IFN-γ-treated BMDM (relative to mock). Log_2_ fold change values were calculated as described for (A).
(EPS)

S4 Fig IFN up-regulates the expression of EVL/ miR-342. (A) IFN-γ or -β effects on mature miR-342-5p abundance in BMDM 7 h or 24 h after treatment. Values are normalised to mock treated cells. Data are mean +/− SEM (*n* = 3), * *p* ≤ 0.05. (B) IFN-γ or -β effects on mature miR-155 abundance in BMDM 7 h or 24 h after treatment. Values are normalised to mock treated cells. Data are mean +/− SEM (*n* = 3), * *p* ≤ 0.05, ** *p* ≤ 0.01. (C) *EVL* and pri/pre-miR-342 RNA transcript synthesis and abundance in BMDM following IFN-γ stimulation (10 U/ml). (D) EVL transcript synthesis (relative to Mock infected cells) in wild-type or TYK2^-/-^ BMDM 60 to 90 and 360 to 390 min after MCMV infection. (E) Pre-miR-342 synthesis (relative to Mock infected cells) in wild-type or TYK2^-/-^ BMDM 60 to 90 and 360 to 390 min after MCMV infection.
(EPS)

S5 Fig IFN directly regulates EVL/ miR-342 transcription. (A) Predicted ISRE and IRF7 binding sites in EVL promoter derived from the software Toucan. (B) Chromatin immunoprecipitation analysis of STAT1 and IRF1 transcription factor binding to *ACTB* (negative control), *CXCL10* (positive control) or the *EVL* promoter in BMDM cultured with IFN-γ (10 U/ml) for 2 h (top) or 24 h (bottom). Data are representative of multiple independent experiments. (C) Primer sequences used to amplify murine ACTB, CXCL10 or EVL promoter DNA isolated by Chromatin Immunoprecipitation.
(EPS)

S6 Fig *SREBF2* siRNA and miR-342-5p effects on cholesterol pathway transcript abundance. *SREBF2* siRNA (upper panel) and miR-342-5p (lower panel) effects on cholesterol pathway transcript abundance in pMEF. Data are mean +/− SEM (*n* = 6), ** *p* ≤ 0.01.
(EPS)

S7 Fig MiR-342-5p targets Srebf2 and miR-33 and regulates intracellular cholesterol by multiple mechanisms. (A and B) Effects of miR-342-5p on relative *SREBF1* or *SREBF2* RNA abundance in mouse and human cells. * *p* ≤0.05, ** *p* ≤0.01 (*n* > 2). (C) IFN-γ or -β effects on mature miR-33 abundance in BMDM 7 h or 24 h after treatment. Values are normalised to mock treated cells. Data are mean +/− SEM (*n* = 3), * *p* ≤ 0.05, ** *p* ≤ 0.01. (D) Schematic showing relationship between LXR regulated transcripts ABCA1 and ABCG1, miR-33 and miR-342-5p. (E) MiR-342-5p effect on miR-33-5p and miR-33-3p abundance in resting or T0901317 treated BMDM.
(EPS)

S8 Fig MiR-342-5p regulates intracellular cholesterol by multiple mechanisms. (A) Quantitation of miR-342-5p effects on total intracellular cholesterol in NIH/3T3 fibroblasts. Data are mean +/− SEM (*n* = 3) * *p* ≤ 0.05. (B) Quantitation of miR-342-5p inhibitor effects on total intracellular cholesterol in NIH/3T3 fibroblasts. Data are mean +/− SEM (*n* = 2). (C) Reduction of Mouse *DHCR7* 3’UTR reporter expression by miR-342-5p. Data are mean +/− SEM (*n* = 3). (D) MiR-342-5p reduces LDLR mRNA abundance in NIH/3T3 cells. Data are mean +/− SEM (*n* = 3), ** *p* < 0.01.
(EPS)

S9 Fig MiR-342-5p regulation of MCMV in vitro and in vivo. (A) Effects of IFN-regulated miRNA on MCMV-GFP replication. Data are normalised to values obtained with non-targeting siRNA and are mean +/− SEM (*n* = 2). (B) Effects of IFN-γ treatment (100 pg/ml for 6 h) on MCMV-GFP replication in negative control transfected NIH/3T3 cells. *N* = 3, *p ≤ 0.05. (C and D) MCMV titre (4 dpi) in Spleen (C) and Liver (D) of BALB/c mice treated with 10 μg or 40 μg miR-342-5p or control miRNA. Data are titre in organ of individual mouse (*n* = 6). *p ≤ 0.05, **p ≤ 0.01. (E) Effects of *AKT1* siRNA miRNA on MCMV-GFP replication. Data are normalised to values obtained with nontargeting siRNA and are mean +/− SEM (*n* = 3). **p ≤ 0.01. (F) Effects of *HMGCR*, *IDI1*, *SC4MOl*, and *DHCR7* siRNA on the abundance of their respective target RNAs in MEF transfected for 48 h. Data are mean +/− SEM (*n* = 3). **p ≤ 0.01.
(EPS)

S10 Fig MiR-342-5p does not affect cell viability. (A and B) Analysis of miR-342-5p effects on cell viability. Primary MEFs (A), 3T3, MRC-5 and HeLa (B) cells were transfected with the indicated doses of control siRNA/ miRNA or miR-342-5p mimics 48 h before analysis using the CellTiter-Blue viability assay. (C) Schematic showing sterol biosynthesis pathway entry points for metabolites used in metabolic rescue experiments.
(EPS)

S11 Fig Schematic showing IFN-activated 25-HC and miR-342-5p pathways regulating sterol metabolism. (EPS)

S1 Methods Extended descriptions of materials and methods. (DOCX)

S1 Table MiR-342-5p is predicted to target the 3’UTR of SREBF2 and multiple cholesterol pathway transcripts. The database TargetScan was queried using mouse Entrez gene IDs for cholesterol biosynthesis pathway members. Target predictions for mouse miRNAs were then tabulated.
(DOCX)

S2 Table MiR-342- 5p is predicted to target Herpesvirus but not Influenza virus transcripts. MiRanda version 3.0 was used to scan viral coding sequences for predicted miRNA binding sites. Results were extracted as key-value pairs and sorted according to total score and free energy.
(DOCX)

## Abbreviations

BMDM: bone marrow-derived macrophage
ChIP: chromatin immunoprecipitation
CH25H: cholesterol 25-hydroxylase
DMAPP: isomer dimethylallyl diphosphate
EVL: ena-vasodilator stimulated phosphoprotein
FOH: farnesol; GGOH, geranylgeraniol
HβCD: (2-Hydroxypropyl)-β-cyclodextrin
HCMV: human cytomegalovirus
HSV1: Herpes Simplex virus 1
IFN: interferon
IFN-β: interferon beta
IFN-γ: interferon gamma
IFNAR1: interferon alpha receptor 1
IPP: isopentyl diphosphate
IRF: interferon regulatory factor
LXR: liver x receptor
MCMV: murine cytomegalovirus
MEV: mevalanolactone
MOI: multiplicity of infection
pMEF: primary murine embryo fibroblasts
Q-RT-PCR: quantitative reverse transcription polymerase chain reaction
siRNA: small interfering RNA
SQL: squalene
TYK2: tyrosine kinase 2
25-HC: 25-hydroxycholesterol
