# Supplementary material for: An Interferon Regulated MicroRNA Provides Broad Cell-Intrinsic Antiviral Immunity through Multihit Host-Directed Targeting of the Sterol Pathway
Source: PLoS Biol. 2016 Mar 3;14(3):e1002364. doi: 10.1371/journal.pbio.1002364 (PMC4777525; doi:10.1371/journal.pbio.1002364)
Supplement: S1 Methods — (DOCX) [file pbio.1002364.s013.docx]

# Supplementary Experimental Procedures

# Mice

C57BL/6 mice were housed in the specific pathogen-free animal facility at the University of Edinburgh. BALB/c mice were housed in the specific pathogen-free animal facility at the Institut d’Investigacions Biomèdiques August Pi i Sunyer, Barcelona, Spain. CH25H^-/-^ (B6.129S6-Ch25h^tm1Rus^/J) mice were purchased from Charles River (Margate, UK) and housed in the specific pathogen-free animal facility at the University of Edinburgh. All procedures were carried out under project and personal licences approved by the Secretary of State for the Home Office, under the United Kingdom's 1986 Animals (Scientific Procedures) Act and the Local Ethical Review Committee at Edinburgh University. In Spain, all procedures involving animals and their care were approved by the Ethics Committee (protocol number CEEA 308/12) of the University of Barcelona and were conducted in compliance with institutional guidelines as well as with national (Generalitat de Catalunya decree 214/1997, DOGC 2450) and international (Guide for the Care and Use of Laboratory Animals, National Institutes of Health, 85-23, 1985) laws and policies.

## Cell Propagation and Culture

Bone marrow-derived macrophages were isolated and grown in DMEM (Lonza, Vervier, Belgium) supplemented with 10% Fetal Bovine Serum (Lonza, Vervier, Belgium), 2mM L-Glutamine, 50U/ml penicillin and streptomycin and CSF-1 derived from L929 cells (10% conditioned medium).

NIH/3T3 cells were obtained from LGC Standards (Teddington, UK) and cultured in DMEM (Lonza, Vervier, Belgium) supplemented with 10% Bovine Serum (Lonza, Vervier, Belgium), 2mM L-Glutamine and 50U/ml penicillin and streptomycin.

HeLa cells were cultured in DMEM (Lonza, Vervier, Belgium) supplemented with 5% Fetal Bovine Serum (Lonza, Vervier, Belgium), L-Glutamine and 50U/ml penicillin and streptomycin.

A549 cells were cultured in DMEM (Lonza, Vervier, Belgium) supplemented with 10% Fetal Bovine Serum (Lonza, Vervier, Belgium) and 2mM L-Glutamine.

Madin Derby Canine Kidney (MDCK) cells were grown in DMEM, 10% Fetal Bovine Serum (Lonza, Vervier, Belgium) and 2mM L-Glutamine.

MRC-5 cells were cultured in EMEM (Lonza, Vervier, Belgium) supplemented with 10% Fetal Bovine Serum (Lonza, Vervier, Belgium), L-Glutamine, Non-Essential Amino Acids and 50U/ml penicillin and streptomycin.

Mouse embryo fibroblasts (MEFs) derived from the embryos of timed pregnant C57BL/6 mice on day 14-17 of gestation, were cultured in Dulbecco’s minimum essential medium (DMEM) supplemented with 10% FCS, 2mM glutamine and 50U/ml penicillin and streptomycin. MEFs were used at passage 3 post-isolation for experiments.

Stat1^-/-^ MEF were provided by B. Strobl (Institute of Animal Breeding and Genetics, Vienna, Austria) and were grown in DMEM, 10% Fetal Bovine Serum (Lonza, Vervier, Belgium) and 2mM L-Glutamine.

RAW264.7 cells were obtained from LGC Standards (Teddington, UK) and cultured in DMEM supplemented with 10% Fetal Bovine Serum (Lonza, Vervier, Belgium) , 50U/ml penicillin and streptomycin and 2mM glutamine.

# Treatment of cells with IFN or LPS

Murine recombinant IFN gamma (IFN-γ) (Perbio Science) and IFN-β (Stratech, UK) were diluted in complete medium and were added to cells at a final concentration of 10U/ml or 25U/ml respectively. Lipopolysaccharides from Escherichia coli 026:B6 (Sigma-Aldrich, UK) were reconstituted in SPBS (1mg/ml) and added to BMDM at a final concentration of 100ng/ml.

# Reporter Viruses and Viral Plaque Assay

The construction of the GFP-encoding Murine Cytomegalovirus (MCMV-GFP, originally named: pSM3fr-rev) used in this study was previously described [1]. The virus was propagated in mouse NIH-3T3 fibrobasts. The amount of infectious MCMV present in the viral stock was quantified by plaque assay on p53^-/-^ MEF monolayers in 48-well plates. The construction of the HCMV-GFP (AD169-GFP) has been previously described [2]. HCMV-GFP was propagated and titred by plaque assay in MRC-5 (lung fibroblast) cells. HSV-1-eGFP (C12) was propagated and titred by plaque assay in Vero cells. A/WSN/33 (H1N1) influenza virus was propagated and titred in MDCK cells.

# Assay for GFP-virus growth

MiRNA mimics or siRNA were transfected into cells at a final concentration of 25nM (unless otherwise indicated) in 0.4% (NIH/3T3, pMEF), 0.2% (MRC-5) or 0.1% (HeLa) DharmaFECT 1 (Thermo Fisher Scientific) with 1.5x10^4^ cells (pMEF) or 3x10^4^ cells (NIH/3T3) per well in a 96 well plate. After 48h or 72h, virus (MCMV MOI = 0.0025, HCMV MOI = 0.5 and HSV MOI = 0.5) was added to cells in 25µl normal (10% serum) medium for 1h. After the adsorption period, virus was removed and 150µl normal medium added to all wells. For the analysis of miR-342-5p inhibitor effects on virus replication, medium containing 3% delipidised serum (Bovine Serum, Lipid Depleted (Part number: S181L), VWR, UK) was used. After infection, the fluorescence in each well was measured using a POLARstar OPTIMA plate reader (BMG Labtech) according to manufacturers recommendations. Virus replication slopes over the linear phase were calculated and then normalized to control transfected wells. The effects of the miR-342-5p mimic on virus growth were normalised to the effects of RISC-free siRNA or C.*elegans* miRNA (Thermo-Fisher Scientific) as indicated whilst the effects of miR-342-5p ZEN-AMO or 2'OMe/LNA-PS inhibitors (IDT, USA) were normalised to a matched negative control RNAs. The mean replication slope for replicates was then used for data analyses.

# BMDM IFN-γ treatment, RNA labelling and isolation

Incorporation of 4-thiouridine (Sigma) into newly-transcribed RNA was undertaken as described by Dölken *et al.* [3]*.* In brief, at time zero medium was aspirated from all plates and 15ml of pre-warmed medium containing IFN-γ or normal medium was added to the cultures. RNA labelling in BMDM during the first 30 minutes of the time course was undertaken by addition of 200µM 4-Thiouridine to the medium of appropriate plates at this time. After 30 minutes, to end the RNA labelling period, terminate transcription and lyse the cells, medium was aspirated from the labelled BMDM and replaced with 4mls of RLT lysis buffer (Qiagen). In parallel, 10mls of medium from the next BMDM cultures to be labelled was added to an appropriate volume of 4-thiouridine, mixed and immediately added back to the plate. BMDM cultures were then returned to the incubator. The above cycle of 4-thiouridine addition to BMDM cultures and transcriptional termination was repeated at 30 minute intervals until the end of the time course.

Total RNA was isolated using an RNeasy Midi kit (Qiagen) according to manufacturers instructions, quantitated using a Nanodrop (Thermo Scientific) and integrity was confirmed using an Agilent Bioanalyser (Agilent UK). Newly transcribed RNA (ntRNA) was then isolated as described in Dölken *et al.* [3] and again quantitated using a Nanodrop.

# Newly transcribed RNA labelling for Microarray Analysis

Processing of ntRNA samples (100ng) for hybridisation to Affymetrix Mouse Gene 1.0 ST arrays was undertaken according to manufacturers instructions (Affymetrix). Hybridisation, washing, staining and scanning of the arrays were also undertaken following standard Affymetrix protocols.

# Microarray Data Analysis

After scanning and data capture, open-source R based software ‘Bioconductor’ was used to implement all quality control and statistical analyses. In brief, to ensure array quality and consistency, data distribution comparisons, all-against-all sample correlations and per-gene measurement precision calculations were undertaken. Primary microarray data are available from the National Centre for Biotechnology Information Gene Expression Omnibus (GEO) under Super-Series accession number GSE42505 (Sub-Series numbers GSE63290) and outputs from these analyses are presented in supporting data.

The RMA method was then used for normalization, background correction, and probe set summation. To reduce the dataset for further analysis and enrich for reliably detected genes, values for median and 2x the Median Absolute Deviation (2xMAD) for negative control probesets were calculated for each array. Intensity values for each probe on each Total RNA array were then compared with their array specific 2xMAD value and were retained if the intensity was equal to or greater than this value. After filtering and removal of all designated Affymetrix control probesets, 12,472 probes were retained for statistical analyses.

  The objective of this study was to capture dynamic changes over time in treated versus untreated samples. Since conventional clustering algorithms do not typically assign significance to differential expression of two conditions over time (on an individual gene basis) the MaSigPro algorithm was used [4]. This algorithm allowed the measurement of a difference between treated and untreated samples in relation to the effects of time and provided a list of genes with p values for use in subsequent analyses. In brief, a 4th-order polynomial model was fitted to the total OR ntRNA time course data from the 12,472 gene probes identified above. Significant differences for each gene between IFN-γ and control time courses were then identified through a step-wise 2-ways backward goodness-of-fit regression at p≤0.05 and r^2^≥0.9. The output of this analysis was 2086 (ntRNA) or 4482 (Total RNA) probes with notable differential profiles of expression across the 8 hours of IFN-γ stimulation.

Heat-maps showing significant and non-significant temporal alterations in sterol biosynthesis pathway-related transcripts in IFN-γ treated BMDM (relative to mock) were produced using the statistical software R. This produced an image in which a column represents 1 time point and row represents 1 gene. In the data presented, each column represents a time period and each row one gene. Gene expression was shown as a pseudo-colour – blue = decrease, red = increase. Log fold change values were calculated by subtracting the mock from the IFN-γ treated signal value.

Q-RT-PCR Analysis

# Q-RT-PCR analysis of cholesterol pathway genes using UPL

The Universal Probe Library was purchased from Roche Applied Science (Roche Diagnostics Ltd. Burgess Hill, United Kingdom). Primer design and probe selection for each gene were undertaken using the Universal Probe Library Assay Design Centre and were as follows: Actb UPL Probe#63 primer reverse: 3’-ctaaggccaaccgtgaaaag-5’ primer forward: 5’-accagaggcatacagggaca-3’, Hmgcr UPL Probe#78 reverse: 3’-tgattggagttggcaccat-5’, forward: 5’-tggccaacactgacatgc-3’, Hmgcs1 UPL Probe#41 reverse: 3’-cagggtctgatcccctttg-5’, forward: 5’-cagagaactgtggtctccaggt-3’, Mvd UPL Probe#73 reverse: 3’-tggcgtcagtgaacaacttc-5’, forward: 5’-acctgagccagggtataggc-3’, Nsdhl UPL Probe#9 primer Reverse: 3’-tgcagctctaggtggaaagg-5’, primer forward: 5’-gaacgtccagaaagggattg-3’, Sqle UPL Probe#12 primer reverse: 3’-gcctctcagaatggtcgtct-5’, primer forward 5’-cgcatctcccagaataagga-3’, Ebp UPL Probe#67 primer Reverse: 3’-tgtcctacagcttgtggtgtct-5’, primer forward 5’-aataaacggggtggcctatc-3’. For each sample, cDNA was synthesised from 1µg total RNA using a SuperScript® III First-Strand Synthesis kit (Invitrogen, UK) according to the manufacturers recommended protocol. CDNAs were then diluted 1/10 in RNAse free water prior to the Q-RT-PCR reaction. For each gene, a probe-primer mix was obtained by combining (per reaction): 0.7µl RNase-free H20, 0.2µl UPL probe (10µM), 0.05µl forward primer (100µM) and 0.05µl reverse primer (100µM). A master mix was obtained by adding 1µl of the probe-primer mix to 3µl RNase-free water and 5µl 2x FastStart Universal Probes Master Science (Roche Diagnostics Ltd. Burgess Hill, United Kingdom). Finally, 1µl of cDNA (per reaction) was combined with 9µl of the master mix in white LightCycler^®^ 480 384 Multiwell Plates using a Freedom Evo-2 150 liquid handling robot (Tecan Trading, Switzerland). To control for genomic DNA contamination, reactions containing RNA from the original sample were analysed in parallel. All samples were then incubated at 95^0^C for 10 minutes followed by 45 cycles of: denaturation at 95^0^C (10 seconds), annealing at 60^0^C (30 seconds) and extension at 72^0^C for 1s. Following data-capture, Roche LightCycler480 software v1.5 was used to calculate differences in transcript abundance. Sample size in this screen was n=3 and Welch's t test was used to test differences in mean transcript abundance between mimic and C. *elegans* miRNA treated controls. The limited statistical power of these tests due to small group sample sizes was recognized. Significant results were interpreted as notable changes to be interpreted or validated in context of other independent findings.

# Q-RT-PCR library analysis of complete sterol pathway

All probe-primer sets were purchased from Integrated DNA Technologies (IDT, USA). Quanta ToughMix (Quanta, 95123) 1-step qPCR master mix was used for all the qRT-PCR assays. For each sample, the Q-RT-PCR was performed in a 10µl volume using 30ng of RNA in 96-well non-skirted, white PCR plates (Thermo Scientific) and flat PCR cap strips (Thermo Scientific). To measure relative gene expression from total cellular RNA, FAM-labelled gene assays for host genes were combined in duplex with a VIC-labelled GAPDH assay (Mm99999915_g1) for normalisation as follows: 5µl TuffMix, 1µl Probe-primer, 0.17µl GAPDH-VIC, 1.83µl H2O and 2µl input RNA. Reverse transcription was undertaken for 10 minutes at 50° C after, which an incubation at 95° C for 1 minutes was used to activate the RNA polymerase. Samples were then subject to 37 cycles of amplification (including annealing and primer extension phase at 60° C for 60 seconds and a short denaturation at 95° C for 10 seconds). Expression levels were measured with a Stratagene Mx3000P qPCR machine (Agilent) and fold changes were determined by the ΔΔCt method using the MxPro software package.

# PCR Analyses of individual genes

Taqman Primer probe sets were purchased from Applied Biosystems, Warrington, UK (Mouse Assay ID: HMGCS1: Mm01304569_m1, HMGCR: Mm01282499_m1 MVD: Mm00507014_m1 SQLE: Mm00436772_m1 SREBF1: Mm00550338_m1, SREBF2: Mm01306292_m1, ABCA1: Mm00442646_m1 , ABCG1: Mm00437390_m1 , ACTB: Mm00607939_s1, Human Assay ID: ACTB: Hs99999903_m1, SREBF2: Hs01081784_m1). For each sample, Q-RT-PCR was performed in 10µl volumes using 96-well Non-Skirted, White PCR Plates (ABgene, UK) and MicroAmp Optical Caps (Applied Biosystems, UK). For 1 reaction, 2µl of diluted total RNA samples (20ng) was added to 2.5µl of qScript One-Step Fast qRT-PCR (Low ROX) master-mix, 0.5µl qScript Reverse Transcriptase (Quanta Biosciences, USA), 0.5µl of Taqman primer/prob set (Applied Biosystems, UK) and 4µl RNase-free H_2_O. Reverse transcription was undertaken for 5 minutes at 50°C after which an incubation at 95°C for for 30 seconds was used to activate the RNA polymerase. Samples were then subject to 40 to 45 cycles as follows (combined annealing and primer extension phase at 60°C for 25 seconds and a short denaturation at 95°C for 3 seconds). Stratagene MXPro software was used to analyse the data. Threshold determinations and differences in transcript abundance relative to *ACTB* were automatically performed by software for each reaction.

# PCR Analysis of mature miRNA

For the purposes of mature miRNA detection, total RNA from *in vitro* tissue culture experiments was isolated using a Qiagen miRNeasy kit according to manufacturers recommendations (Qiagen, USA). MicroRNA assays used (all purchased from Quanta Biosciences, USA) were as follows: mmu-miR342-5p (MMIR-0342-5P), mmu-miR-342-3p (HSMIR-0342-3P), mmu-miR-155-5p (MMIR-0155), mmu-miR-33 (HSMIR-0033A) and SNORD47 (MM-SNORD47). Two-step PCR analyses were undertaken as per manufacturers protocols using the qScript microRNA cDNA Synthesis Kit (Quanta Biosciences, USA) followed by relative quantitation of miRNAs using the [PerfeCTa® SYBR® Green SuperMix, Low ROX](http://www.quantabio.com/product.php?base_id=95056) (Quanta Biosciences, USA). Stratagene MXPro software was used to analyse the data. Threshold determinations and differences in transcript abundance relative to the SNORD47 small RNA control were automatically performed by software for each reaction.

**PCR Quantitation of murine SREBF2 transcript copy number**

Two-step PCR analyses of SREBF2 copy number in total RNA samples from transfected cells were undertaken as per manufacturers protocols using qScript cDNA SuperMix (Quanta Biosciences, USA) followed by absolute quantitation of *SREBF2* copy number using PerfeCTa SYBR Green FastMix, Low ROX (Quanta Biosciences, USA). A standard curve was constructed by serial dilution of a plasmid containing the SREBF2 amplicon sequence. The pEX-A2 plasmid containing the sequence was synthesised by MWG-Eurofins.

| **SREBF2 sequence** | ACAGTGATGTGGACTTGAAAATTGATGACTTTAACCAGAATGTCCTTCTGATGTCTCCGCCGGCCTCCGACTCCGGGTCCCAGGCCGGCTTCTCTCCCTATTCCATTGACTCTGAGCCGGGCAGCCCTCTGCTGGATGACGCAAAGGTCAAGGATGAACCGGACTCTCCTCCTGTGGCACTGGGCATGGTGGACCGCTCTCGAATCCTCTTATGTGTCCTCACCTTCCTGGGCCTCTCCTTTAACCCCTTGACTTCCTTGCTGCAGTGGGGAGGGGCCCACAACACTGACCAGCACCCATACTCAGGCTCGGGCCGCAGTGTACTGTCACTGGAGTCAGGTGCTGGGGGCTGGTTTGACTGGATGGTTCCAACTCTCCTCCTGTGGCTGGTAAATGGTGTGATTGTCTTGAGCGTCTTTGTGAAGCTGTTGGTCCACGGGGAGCCGGTGATTCGCCCACACTCACGCCCCTCAGTCACCTTCTGGAGACACCGGAAGCAGGCAGACCTAGACCTCGCCAAAGGTGATTTCGCAGCTGCTGCTGCCAACCTACAAACCTGCTTATCCGTGCTGGGCCGGGCGCTGCCCACCTCCCGCCTGGACTTGGCCTGCAGCCTCTCCTGGAATGTGATCCGCTACAGCCTGCAGAAGCTGCGCCTGGTACGCTGGTTACTCAAGAAGGTCTTCCAGCGCTGGCGGGCTACTCCCGCC |
| --- | --- |
| **SREBF2 For** | 5'-GACCGCTCTCGAATCCTCTTATGTG-3' |
| **SREBP2 Rev** | 5'-GTTTGTAGGTTGGCAGCAGCA-3' |

Primers for the amplification of the SREBF2 sequence were obtained from Ma *et al.* (2014) [5]. Stratagene MXPro software was used to analyse the data.

# Transfection of mimics, inhibitors and siRNA

For transfection, miRIDIAN microRNA mimics, *Caenorhabditis elegans* miRNAs and ‘RISC-free’ siRNA were purchased from Dharmacon RNAi Technologies, Thermo Fisher Scientific (Lafayette, USA). For gene knockdown experiments mimics and siRNA were transfected into NIH/3T3, pMEF or MRC-5 cells at final concentrations of between 25nM and 50nM in 0.4% (pMEF/ 3T3) or 0.15% (A549) DharmaFECT 1 (Thermo Fisher Scientific) with 1x10^5^ cells per well in a 24 well plate. After 48 hours, medium was aspirated from wells and RNA isolated using a Qiagen RNeasy Plus kit according to manufacturers instructions. For experiments in BMDM or RAW cells: miR-342-5p microRNA mimic, miR-342-5p ZEN-AMO inhibitor, miR-342-5p 2’OMe/LNA-PS inhibitor, NC-5 non-targeting control miRNA, ZEN-AMO non-targeting control and 2’OMe/LNA-PS non-targeting control were kindly gifted by Integrated DNA Technologies [6] and transfected at a final concentration of 25nM (unless otherwise stated) (Leuven, Belgium). Mimics, inhibitors and siRNA were transfected into BMDM or RAW cells using Viromer Blue (Lipocalyx, Germany) as per manufacturers recommendations.

# Administration of small RNAs to mice

For *in vivo* experiments, miR-342-5p microRNA mimic and NC-5 non-targeting control miRNA were obtained from Integrated DNA Technologies (Leuven, Belgium). miRNA were administered by an intra-peritoneal injection route as previously described [7]. In brief, each mouse received 10 or 40µg in total of the small RNAs. RNAs were complexed with Transit-TKO and were delivered by injection of 2µg, 4µg and 4µg or 8µg, 16µg and 16µg of the RNA respectively on days 1,2 and 4 of the experiment. For infection, mice were injected with 1x10^6^ PFU MCMV in SPBS on day 3 of the experiment. Mock-infected animals were injected in an identical manner with SPBS only. To reduce the potential effects of insulin on cholesterol metabolism, mice were starved for 6h on day 7 of the experiment and then culled. Heart, lungs, liver, spleen and kidney were collected and snap frozen for subsequent analysis of MCMV titre.

# Transfection of 3’UTR luciferase reporter plasmids and miRNA

To assess the specificity of miRNA targeting, psiCHECK-2 vectors (Promega) containing a multiple cloning site (MCS) downstream of an SV40 promoter-driven renilla luciferase gene and a constitutively-expressed firefly luciferase gene were used. For all genes tested*,* regions from the 3’UTR of the gene were synthesized and sub-cloned into the 3’UTR MCS of the psiCheck2 renilla luciferase by Eurofins MWG Operon (Ebersberg, Germany). In the case of *SREBF2* and *IDI1*, UTRs were synthesized containing predicted miR-342-5p targets or corresponding mutant fragments (472bp) lacking the miRNA seed region. For *DHCR7* and *SC4MOL*, regions from the 3’UTR containing intact miR-342-5p targets only were synthesized and sub-cloned into the psiCheck2 plasmid. Finally, for mouse *SREBF1* (whose 3’UTR does not contain a predicted miR-342-5p target) a 768bp region (>92%) of the 3’ UTR was synthesized and sub-cloned into the psiCheck2 vector. To assess miRNA target specificity, the miR-342-5p mimic or C. *elegans* negative control miRNA were reverse transfected into HEK-293T cells at final concentration of 50nM with 50ng per well of either wild-type or mutant luciferase reporter in 0.015% DharmaFECT DUO. After 24h, luciferase expression in the transfected cells was measured using a dual-luciferase reporter assay kit (Promega, UK) and a BMG polar star plate reader according to manufacturers recommendations. Measurements between different transfections were rendered comparable through control-well normalisation. Briefly, individual firefly luciferase values were numerically corrected by the amount they differ from the median of all firefly luciferase wells. The same correction was then also applied to renilla luciferase luminescence measurements. The outcome if this was that all values for luminescence were relative to the Firefly luciferase and differences in transfection efficiency between wells were minimised.

# Western blot analysis

Cells were washed with PBS and re-suspended in whole-cell lysis buffer (50 mM Tris-HCl, pH 7.5, 100 mM NaCl, 1% NP40, 1mM phenylmethanesulfonyl fluoride, 1µl Protease Inhibitor Cocktail (Sigma, UK) and 1x complete protease inhibitor tablet (Roche, UK)). Cell lysates were centrifuged at 4°C for 10 min and supernatants stored at -20°C. Protein concentration was measured using a BCA assay (Thermo Scientific). For Western blotting, proteins were separated by SDS-PAGE, transferred to Immobilon-FL membranes (Millipore), and probed with rabbit polyclonal anti-SREBP2 (Novus Biologicals, NBP1-39687, 1:1000) and rabbit anti-β-actin (Cell Signalling, 4970, 1:2500) diluted in PBST (0.1% Tween20). The secondary IR-800 anti-rabbit (Cell Signalling, 5151, 1:10,000) antibody was diluted in Odyssey blocking buffer (0.1% Tween20, 0.01% SDS). For probing and visualization, the Odyssey protocol (LI-COR) was followed as per manufacturers recommendations.

# Treatment of cells with 25-Hydroxycholesterol or (2-Hydroxypropyl)-β-cyclodextrin

25-Hydroxycholesterol (25-HC, Sigma, H1015) was dissolved in 100% Ethanol (1000x stock, 20mM) and stored at -20^0^C under argon in 2ml opaque tubes with gasket screw-top lids. The powder (2-Hydroxypropyl)-β-cyclodextrin (HBCD) (Sigma, H107) was dissolved in medium at 37^0^C just before use.

# Measurement of total cholesterol concentration by enzymatic assay

To harvest adherent cells, medium was aspirated and the monolayer washed 2x with an excess of ice-cold sterile PBS. Cells were scraped, centrifuged and re-suspended in 200µl ice-cold PBS, snap frozen and stored at -80^0^C. To extract cholesterol, cell lysates were thawed, 20µl was taken for protein quantitation and the remaining 180µl was added to 2mls RNase/DNase-free water in a 6ml Teflon-capped tube. To each tube was added 4.4mls Chloroform/ Methanol (1;1, V:V) and samples were vortexed until phases were well mixed. Samples were centrifuged at 1360g for 10 minutes at 4^0^C and lower organic phase removed into a new glass tube. Finally, the organic phase was evaporated and lipids dried under Nitrogen with gentle heating and tubes stored at -20^0^C. Prior to analysis, lipids were dissolved in 100% ethanol at 37^0^C. Total Cholesterol quantitation was carried out using an Amplex Red Cholesterol Assay Kit as per manufacturers recommendations (Invitrogen, UK).

# Analysis of Sterols by Mass Spectrometry

Cells were washed with cold PBS (3 mL), re-suspended in ice cold PBS (1.6 mL), scraped into polypropylene tubes, centrifuged and re-suspended in cold SPBS (1 mL). Two hundred microliters of the cell suspension was then removed and snap-frozen for PCR analysis of DNA copy number (for use in normalisation). The suspension was then centrifuged once more and cold absolute ethanol (1 mL) added to the cell pellet. After further centrifugation, isotope-labelled internal standards (2 ng 24R/S-[^2^H_7_]hydroxycholesterol, 2 ng 22R-[^2^H_7_]cholest-4-en-3-one, 0.2 ng 7α,25-[^2^H_6_]dihydroxycholesterol and 2 μg [^2^H_7_]cholesterol) were added in absolute ethanol (1.1 mL) to the supernatant and ultrasonicated for 5 min. This solution was diluted to 70% ethanol with water followed by further ultrasonication and centrifuged at 3000 g at 4 ^o^C for 30 min.

Sterols in the cell extract were separated from oxysterols on a Certified Sep-Pak C_18_ 200 mg column (Waters). The column was washed with absolute ethanol (4 mL) and conditioned with 70% ethanol (6 mL). The cell extract in 70% ethanol (3 mL) was added to the column. Oxysterols were eluted in the flow-through and a 4 mL wash with 70% ethanol. After a further 2 mL wash with 70 % ethanol, cholesterol and sterols of similar polarity were eluted in absolute ethanol (2 mL). Each fraction was dried down and re-constituted in propan-2-ol (100 μL).

Sterols and oxysterols were analysed using enzyme-assisted derivatisation for sterol analysis (EADSA) technology [8]. In brief, sterols/oxysterols were oxidised with cholesterol oxidase (0.26 u in 1 mL 50 mM KH_2_PO_4_ buffer) at 37 ^o^C for 1 hr. The reaction was quenched with methanol (2 mL). Glacial acetic acid (150 μL) was added to the solution followed by Girard P hydrazine reagent (150 mg). The mixture was vortexed and left overnight at room temperature in the dark. Excess reagent was removed by solid phase extraction using Oasis HLB 60 mg cartridges (Waters). After washing and conditioning of the cartridges with methanol (6 mL), 10% methanol (6 mL) and 70% methanol (4 mL) the derivatisation solution (~ 3 mL 70% methanol) was added. A re-cycling protocol was adopted where the eluate was diluted first to 35% methanol and then to 17% methanol and at each dilution re-applied to the column. The column was then washed with 10% methanol (6 mL) and sterols/oxysterols eluted in 3 x 1 mL of methanol.

Sterols and oxysterol were analysed by liquid chromatography – mass spectrometry (LC-MS) on an Orbitrap Elite (ThermoFisher) operated at 100,000 resolution (FWHM) with better than 2 mmu mass accuracy in the MS mode as described previously [8]. Sterols and oxysterols were identified by comparison of *m/z*, retention time and MS^n^ fragmentation with reference standards. Quantification was by stable isotope-dilution.

# Computational prediction of miRNA target binding sites in sterol-related genes

For the prediction of potential microRNA targets in genomic 3’ UTR regions the database TargetScan was used [9] . For the prediction of microRNA targets in 5’UTR, coding regions and 3’UTRs the database miRWalk was used [10].

# Computational prediction of miRNA target binding sites in virus genes

MiRanda version 3.0 was used to scan viral coding sequences with the —strict option to exclude mismatches or wobble pairs within the seed region of the microRNA. The mature microRNA sequence used was obtained from miRBase v21 (mmu-miR-342-5p) [11]. The viral coding sequences were obtained from GenBank via NCBI for the following accessions: MCMV (NC_004065.1), HCMV (FJ527563.1), HSV1 (X14112.1) and influenza A (A/WSN/33 (H1N1): X14112.1, CY010795.1, CY010794.1, CY010793.1, CY010788.1, CY010791.1, CY010790.1, CY010789.1 and CY010792.1). MiRanda results were extracted as key-value pairs and sorted according to total score and free energy.

# Computational prediction of transcription factor binding sites in Evl promoter from Human and Mouse

To analyse and predict potential Stat1, Irf1 and Irf9 binding sites in the promoter of Human and Mouse EVL/Evl, the open source software Toucan was used [12]. In brief, 1Kb 5’ Cis-regulatory regions upstream of the transcriptional start sites of Human or Mouse Evl were imported into Toucan. Predicted transcription factor binding sites were then identified in these sequences using the MotifLocator algorithm. Position Weight Matrices for this analysis were derived from the TransFac database V7.0 (public) and the background model used was either Mouse or Human DBTSS promoters (0) depending on input sequence. A default stringency threshold of 0.9 was used for all predictions.

# Chromatin Immunoprecipitation and PCR analysis of promoter sequence enrichment.

BMDM were treated with Ifn-γ (10U/ml) for 2, 6 and 24h, fixed with 1% formaldehyde (25°C, 10 min) in PBS and the reaction stopped with 0.125M Glycine. Chromatin immunoprecipitation (ChiP) was performed as described previously. Nuclei were sonicated using a Diagenode Bioruptor (Liege, Belgium) on full power 30s on then 30s off in an icebath for 60min. This produced DNA fragments of <300bp. One hundred to 200µg chromatin was incubated with 4µg rabbit IgG (Santa Cruz, sc-2025), Stat1 antibody (AbCam, Ab2415) or anti-Irf1 antibody (Cambridge Biosciences, A303-376A) and washed, eluted and cross-links reversed. Primers for the amplification of promoter regions (predicted to contain the Stat1 or Irf1 binding sites) from Evl and the positive control gene Cxcl10 were then designed using PrimerBLAST and are provided in Supplementary Figure 2. The amplification of a single product by primer sets was confirmed by melt-curve analysis. Quantitative-PCR using SYBR-green incorporation (Quanta PerfeCTa® SYBR® Green FastMix®, Low ROX) was used to analyse enrichment of sequences relative to input DNA.

# Analysis of EVL promoter activation

Predicted binding sites for transcription factors were identified in a 1KB region of the Human EVL promoter as described above. To assess the importance of a high scoring predicted ISRE sequence and 2 potential Irf7 binding sites, pGL4.1 vectors (Promega, UK) containing a multiple cloning site (MCS) upstream of a firefly luciferase gene were used. A 421bp region from the Human EVL promoter containing the 3 predicted IFN activated transcription factor binding sites was synthesized and sub-cloned into the MCS of the pGL4.1 luciferase plasmid by Eurofins MWG Operon (Ebersberg, Germany). In parallel, corresponding mutants of each individual site (designated: ISRE, proximal Irf7 and distal Irf7) and a mutant in which all predicted sites were mutated (designated: All) were produced. Promoter activation by type 1 IFN was then tested. In brief, 6x10^3^ primary MEF were cultured in each well of 96 well plate overnight. Cells were then co-transfected using JetPRIME (Polyplus, Illkirch, France) with pGL4.1 plasmids containing the promoter of interest and a transfection control plasmid constitutively expressing renilla luciferase (Promega, UK). After 4h, the transfection reagent and medium were removed, fresh medium added to all wells and the cells cultured overnight. On the day of the assay, Ifnb1 (25U/ml) then added to the appropriate wells, cells were cultured for 4h then harvested and Firefly/Renilla luciferase expression assayed using the Dual-Luciferase Reporter Assay System (Promega, UK).

# Metabolite and LXR agonist treatment of cells

After washing, normal medium containing vehicle (Ethanol) or Geranylgeraniol (GGOH) (Sigma G3278), Mevalonolactone (Mev) (Sigma M4667), Farnesol (FOH) (Sigma F203) or Squalene (SQL) (Sigma S3626) was added to the infected wells. T0901317 (Tocris Bioscience, Bristol, UK) was re-suspended in 100% ethanol (5mM stock), diluted in normal medium (50nM final concentration) and cells were treated for 18h.

# Statistical analyses

Unless otherwise stated, a two-sample Welch t-test was used to test statistical significance of results in Microsoft Excel. Prior to parametric testing, a Shapiro-Wilks test was undertaken in R to confirm normal distribution of data. Statistical testing of Q-RT-PCR data from independent experiments (normalised to housekeeping gene ACTB) was undertaken using a one-sample t test in Microsoft Excel. Group sizes (‘n’ numbers) are stated in figure legends.

# Cholesterol Biosynthesis Model

The cholesterol biosynthesis model used in this study was derived from a comprehensive consensus Systems Biology Graphical Notation diagram of the regulation and feedback of cholesterol metabolism [13]. For the purposes of brevity all metabolite names have been replaced with LipidMaps Consortium PubChem Substance ID’s.

1. Angulo A, Ghazal P, Messerle M. The major immediate-early gene ie3 of mouse cytomegalovirus is essential for viral growth. J Virol. 2000;74(23):11129-36. Epub 2000/11/09. PubMed PMID: 11070009; PubMed Central PMCID: PMC113196.

2. Borst E, Messerle M. Development of a cytomegalovirus vector for somatic gene therapy. Bone Marrow Transplant. 2000;25 Suppl 2:S80-2. Epub 2000/08/10. PubMed PMID: 10933196.

3. Dolken L, Ruzsics Z, Radle B, Friedel CC, Zimmer R, Mages J, et al. High-resolution gene expression profiling for simultaneous kinetic parameter analysis of RNA synthesis and decay. RNA. 2008;14(9):1959-72. Epub 2008/07/29. doi: rna.1136108 [pii] 10.1261/rna.1136108. PubMed PMID: 18658122; PubMed Central PMCID: PMC2525961.

4. Conesa A, Nueda M, Ferrer A, Talon M. maSigPro: a method to identify significantly differential expression profiles in time-course microarray experiments. Bioinformatics. 2006;22(9):1096-102. doi: 10.1093/bioinformatics/btl056. PubMed PMID: WOS:000236997600009.

5. Ma K, Malhotra P, Soni V, Hedroug O, Annaba F, Dudeja A, et al. Overactivation of Intestinal SREBP2 in Mice Increases Serum Cholesterol. Plos One. 2014;9(1). doi: 10.1371/journal.pone.0084221. PubMed PMID: WOS:000330240500011.

6. Lennox K, Owczarzy R, Thomas D, Walder J, Behlke M. Improved Performance of Anti-miRNA Oligonucleotides Using a Novel Non-Nucleotide Modifier. Molecular Therapy-Nucleic Acids. 2013;2. doi: 10.1038/mtna.2013.46. PubMed PMID: WOS:000332467100005.

7. Lundberg P, Yang HJ, Jung SJ, Behlke MA, Rose SD, Cantin EM. Protection against TNFalpha-dependent liver toxicity by intraperitoneal liposome delivered DsiRNA targeting TNFalpha in vivo. Journal of controlled release : official journal of the Controlled Release Society. 2012;160(2):194-9. Epub 2011/11/19. doi: 10.1016/j.jconrel.2011.10.034. PubMed PMID: 22094102; PubMed Central PMCID: PMC3321390.

8. Griffiths W, Crick P, Wang Y, Ogundare M, Tuschl K, Morris A, et al. Analytical strategies for characterization of oxysterol lipidomes: Liver X receptor ligands in plasma. Free Radical Biology and Medicine. 2013;59:69-84. doi: 10.1016/j.freeradbiomed.2012.07.027. PubMed PMID: WOS:000318202800008.

9. Agarwal V, Bell G, Nam J, Bartel D. Predicting effective microRNA target sites in mammalian mRNAs. Elife. 2015;4. doi: 10.7554/eLife.05005. PubMed PMID: WOS:000359681800001.

10. Dweep H, Sticht C, Pandey P, Gretz N. miRWalk--database: prediction of possible miRNA binding sites by "walking" the genes of three genomes. J Biomed Inform. 2011;44(5):839-47. doi: 10.1016/j.jbi.2011.05.002. PubMed PMID: 21605702.

11. Kozomara A, Griffiths-Jones S. miRBase: annotating high confidence microRNAs using deep sequencing data. Nucleic Acids Research. 2014;42(D1):D68-D73. doi: 10.1093/nar/gkt1181. PubMed PMID: WOS:000331139800011.

12. Aerts S, Van Loo P, Thijs G, Mayer H, de Martin R, Moreau Y, et al. TOUCAN 2: the all-inclusive open source workbench for regulatory sequence analysis. Nucleic acids research. 2005;33(Web Server issue):W393-6. Epub 2005/06/28. doi: 10.1093/nar/gki354. PubMed PMID: 15980497; PubMed Central PMCID: PMC1160115.

13. Mazein A, Watterson S, Hsieh WY, Griffiths WJ, Ghazal P. A comprehensive machine-readable view of the mammalian cholesterol biosynthesis pathway. Biochem Pharmacol. 2013;86(1):56-66. doi: 10.1016/j.bcp.2013.03.021. PubMed PMID: 23583456; PubMed Central PMCID: PMC3912678.
