# Supplementary material for: An Interferon Regulated MicroRNA Provides Broad Cell-Intrinsic Antiviral Immunity through Multihit Host-Directed Targeting of the Sterol Pathway
Source: PLoS Biol. 2016 Mar 3;14(3):e1002364. doi: 10.1371/journal.pbio.1002364 (PMC4777525; doi:10.1371/journal.pbio.1002364)
Supplement: S1 Table — The database TargetScan was queried using mouse Entrez gene IDs for cholesterol biosynthesis pathway members. Target predictions for mouse miRNAs were then tabulated. (DOCX) [file pbio.1002364.s014.docx]

| **Gene Symbol** | **miR-342-5p** | **miR-342-3p** | **miR-155** |
| --- | --- | --- | --- |
| *SREBF1* | N | N | N |
| *SREBF2* | Y | N | N |
| *HMGCS1* | N | N | N |
| *HMGCR* | N | N | N |
| *MVK* | N | N | N |
| *PMVK* | N | N | N |
| *MVD* | N | N | N |
| *FDPS* | N | N | N |
| *IDI1* | Y | N | N |
| *FDFT1* | N | N | N |
| *SQLE* | N | N | N |
| *LSS* | N | N | N |
| *CYP51* | N | N | N |
| *TM7SF2* | N | N | N |
| *SC4MOL* | Y | N | N |
| *NSDHL* | N | N | N |
| *HSD17B7* | N | N | N |
| *DHCR24* | Y | N | N |
| *EBP* | N | N | N |
| *SC5D* | N | N | N |
| *DHCR7* | Y | N | N |
