# Supplementary material for: An Interferon Regulated MicroRNA Provides Broad Cell-Intrinsic Antiviral Immunity through Multihit Host-Directed Targeting of the Sterol Pathway
Source: PLoS Biol. 2016 Mar 3;14(3):e1002364. doi: 10.1371/journal.pbio.1002364 (PMC4777525; doi:10.1371/journal.pbio.1002364)
Supplement: S2 Table — MiRanda version 3.0 was used to scan viral coding sequences for predicted miRNA binding sites. Results were extracted as key-value pairs and sorted according to total score and free energy. (DOCX) [file pbio.1002364.s015.docx]

| **Virus GenBank ID** | **Hit Sequence** | **ORF Description** | **Total Score** | **Delta G** | **Length** | **Position1** | **Position2** |
| --- | --- | --- | --- | --- | --- | --- | --- |
| **Herpes Simplex Virus 1** | | | | | | | |
| **X14112.1** | **CAA32284.1_70** | **US7 virion glycoprotein I** | **167** | **-27.99** | **23** | **1003** | **1026** |
| **X14112.1** | **CAA32323.1_33** | **UL30 DNA polymerase** | **164** | **-21.639999** | **24** | **3571** | **3595** |
| **X14112.1** | **CAA32310.1_38** | **UL35 capsid protein** | **164** | **-24.440001** | **35** | **95** | **130** |
| **X14112.1** | **CAA32294.1_47** | **UL44 virion glycoprotein C** | **164** | **-24.82** | **21** | **947** | **967** |
| **X14112.1** | **CAA32311.1_39** | **UL36 very large tegument protein** | **161** | **-24.450001** | **23** | **595** | **618** |
| X14112.1 | CAA32311.1_39 | UL36 very large tegument protein | 161 | -22.91 | 19 | 75 | 93 |
| X14112.1 | CAA32305.1_45 | UL42 subunit of replicative DNA polymerase | 154 | -20.200001 | 18 | 1241 | 1262 |
| X14112.1 | CAA32326.1_15 | Ul13 protein kinase | 153 | -25.17 | 31 | 319 | 350 |
| X14112.1 | CAA32307.1_35 | UL32 DNA packaging protein UL32 | 153 | -21.65 | 20 | 1026 | 1047 |
| X14112.1 | CAA32338.1_4 | UL2 uracil-DNA glycosylase | 152 | -20.48 | 18 | 481 | 501 |
| X14112.1 | CAA32276.1_75 | US11 virion protein | 151 | -20.49 | 20 | 66 | 88 |
| X14112.1 | CAA32290.1_58 | UL54 immediate early protein | 150 | -30.440001 | 28 | 617 | 647 |
| X14112.1 | CAA32304.1_44 | UL41 tegument protein; host shut-off factor | 138 | -23.17 | 22 | 1068 | 1089 |
| X14112.1 | CAA32281.1_67 | US4 virion glycoprotein G | 135 | -23.309999 | 20 | 421 | 443 |
| X14112.1 | CAA32294.1_47 | UL44 virion glycoprotein C | 132 | -21.02 | 21 | 165 | 186 |
| X14112.1 | CAA32280.1_66 | US3 protein kinase phosphorylates UL34 protein | 131 | -24.530001 | 24 | 573 | 597 |
| X14112.1 | CAA32284.1_70 | US7 virion glycoprotein I | 130 | -20.110001 | 22 | 630 | 651 |
| X14112.1 | CAA32311.1_39 | UL36 very large tegument protein | 125 | -22.639999 | 26 | 6900 | 6926 |
| X14112.1 | CAA32301.1_54 | UL50 deoxyuridine triphosphatase | 108 | -20.879999 | 27 | 708 | 733 |
| **Human Cytomegalovirus (AD169)** | | | | | | | |
| **FJ527563.1** | **ACL51152.1_78** | **UL83 tegument protein pp65** | **178** | **-28.719999** | **21** | **1082** | **1102** |
| **FJ527563.1** | **ACL51162.1_88** | **UL94 tegument protein UL16** | **173** | **-26.83** | **21** | **712** | **735** |
| FJ527563.1 | ACL51111.1_36 | UL31 DURP family | 166 | -22.65 | 21 | 1608 | 1629 |
| FJ527563.1 | ACL51158.1_84 | UL89 DNA packaging terminase subunit 1 | 164 | -23.18 | 27 | 118 | 145 |
| FJ527563.1 | ACL51216.1_141 | US13 membrane protein | 164 | -21.860001 | 26 | 252 | 277 |
| FJ527563.1 | ACL51155.1_81 | UL86 major capsid protein | 163 | -24.48 | 24 | 1585 | 1606 |
| FJ527563.1 | ACL51116.1_41 | UL36 tegument protein vICA | 162 | -21.620001 | 26 | 213 | 238 |
| FJ527563.1 | ACL51127.1_52 | UL48 large tegument protein | 158 | -20.969999 | 26 | 5027 | 5053 |
| FJ527563.1 | ACL51151.1_77 | UL82 upper matrix protei | 152 | -20.02 | 21 | 1636 | 1659 |
| FJ527563.1 | ACL51212.1_137 | US9 membrane glycoprotein US9 | 152 | -21.219999 | 24 | 347 | 371 |
| FJ527563.1 | ACL51076.1_1 | RL1 protein RL1 | 151 | -21.08 | 26 | 686 | 712 |
| FJ527563.1 | ACL51133.1_58 | UL53 nuclear egress lamina protein | 150 | -20.41 | 18 | 195 | 217 |
| FJ527563.1 | ACL51217.1_142 | US14 membrane protein US14 | 150 | -27.299999 | 24 | 355 | 376 |
| FJ527563.1 | ACL51132.1_57 | UL52 DNA packaging protein | 149 | -20.98 | 21 | 1759 | 1777 |
| FJ527563.1 | ACL51165.1_91 | UL97 tegument serine/threonine protein kinase | 149 | -21.23 | 18 | 602 | 624 |
| FJ527563.1 | ACL51205.1_130 | IRS1, tegument protein IRS1, transcriptional activator; | 149 | -20.65 | 23 | 957 | 982 |
| FJ527563.1 | ACL51237.1_163 | TRS1, transcriptional activator | 149 | -20.65 | 23 | 957 | 982 |
| FJ527563.1 | ACL51076.1_1 | RL1 | 134 | -20.790001 | 22 | 656 | 675 |
| FJ527563.1 | ACL51155.1_81 | UL86, major capsid protein | 134 | -20.129999 | 21 | 2502 | 2526 |
| **Mouse Cytomegalovirus (Smith Strain)** | | | | | | | |
| **NC_004065.1** | **YP_214034.1_26** | **MuHV1_gp026 UL25 (GF1)** | **169** | **-27.66** | **23** | **2705** | **2730** |
| **NC_004065.1** | **YP_214071.1_63** | **MuHV1_gp063 UL69 Similar to HCMV transactivator (E to L)** | **168** | **-26.120001** | **20** | **1315** | **1336** |
| **NC_004065.1** | **YP_214050.1_42** | **MuHV1_gp042 UL38 hypothetical protein** | **164** | **-23.879999** | **22** | **839** | **861** |
| NC_004065.1 | YP_214085.1_77 | MuHV1_gp077 UL83 (pp65) Lower matrix phosphoprotein | 163 | -20.389999 | 18 | 1150 | 1170 |
| NC_004065.1 | YP_214073.1_65 | MuHV1_gp065 UL70 (HP) (34) hypothetical protein | 162 | -21.24 | 21 | 2017 | 2038 |
| NC_004065.1 | YP_214143.1_134 | MuHV1_gp134 US22 family homolog | 159 | -22.459999 | 23 | 983 | 1007 |
| NC_004065.1 | YP_214046.1_38 | MuHV1_gp038 UL32 (pp150) Tegument | 158 | -20.08 | 18 | 1869 | 1888 |
| NC_004065.1 | YP_214129.1_120 | MuHV1_gp120 US22 (GF2); ie2 exon 3, with mRNA terminating at base 187353 | 157 | -21.67 | 22 | 499 | 520 |
| NC_004065.1 | YP_214048.1_40 | MuHV1_gp040 UL35 (GF1) UL25 family homolog | 156 | -21.08 | 20 | 1349 | 1371 |
| NC_004065.1 | YP_214070.1_62 | MuHV1_gp062 hypothetical protein | 156 | -21.26 | 27 | 341 | 368 |
| NC_004065.1 | YP_214070.1_62 | MuHV1_gp062 hypothetical protein | 148 | -21.83 | 23 | 270 | 293 |
| NC_004065.1 | YP_214047.1_39 | MuHV1_gp039 hypothetical protein | 137 | -21.030001 | 25 | 2363 | 2389 |
| NC_004065.1 | YP_214122.1_113 | MuHV1_gp113 UL121 P; Serine-alanine-rich glycoprotein with low homology to HSV-1 ICP0 | 136 | -22 | 22 | 477 | 501 |
| NC_004065.1 | YP_214058.1_50 | MuHV1_gp050 | 134 | -20.92 | 22 | 557 | 579 |
| NC_004065.1 | YP_214068.1_60 | MuHV1_gp060 HSV ICP18.5 homolog [UL28 terminase virion packaging] | 134 | -21.450001 | 24 | 1777 | 1798 |
| NC_004065.1 | YP_214139.1_130 | MuHV1_gp130 US22 family homolog | 132 | -20.190001 | 31 | 1605 | 1636 |
